# High genomic diversity of Vibrio parahaemolyticus from underexplored tropical northern Australia: a baseline for future surveillance

**DOI:** 10.1099/mgen.0.001536

**Published:** 2025-10-27

**Authors:** Mirjam Kaestli, Karen Gibb, Claire E. Hedges, Anna Padovan

**Affiliations:** 1 Research Institute for the Environment and Livelihoods, Charles Darwin University, Darwin, Northern Territory, 0909, Australia; 2 Institute for Marine and Antarctic Studies (IMAS), College of Sciences and Engineering, University of Tasmania, Hobart, Tasmania, 7053, Australia

**Keywords:** northern Australia, One Health, pangenome analysis, pathogenicity island, phylogenetic analysis, TDH-related haemolysin (TRH) variants, *Vibrio parahaemolyticus*, whole-genome sequencing

## Abstract

*Vibrio parahaemolyticus* is an autochthonous marine bacterium that causes gastroenteritis after ingestion of raw or undercooked seafood and, less frequently, wound and tissue infections. Genomic information from northern Australia is scarce, and most sequences to date stem from foodborne outbreaks in southern states. We analysed 24 environmental and 5 clinical isolates (4 wound and 1 gastrointestinal case) collected in the wet–dry tropics from northern Australia and placed them alongside 48 representative public genomes. Core-genome phylogeny showed that the northern Australian strains sit within the broad VppAsia lineage and intermix with Asian and South American isolates, reflecting the limited geographic structuring in the marine environment. A gastrointestinal isolate (RDH3, ST-2901) harboured the *trh2*-positive VPaI-β pathogenicity island (including *vtrAB* and *hlyDBAC* genes) and displayed an A187S change in the TRH2 toxin which was absent in the other TRH-1/-2 sequences of the isolates analysed in this study. Wound isolates lacked *tdh* and *trh* genes, suggesting that the species’ intrinsic virulome may suffice for tissue colonization; notably, one wound strain carried a plasmid with a *Vibrio alginolyticus pilT* copy linked to twitching motility which has been associated with wound infections. It remains to be determined whether this additional *pilT* copy, beyond the core genome copies, confers a selective advantage for tissue colonization. Plasmid diversity amongst clinical isolates and a diseased aquaculture fish, including a putative chimaera plasmid in the gastrointestinal isolate, underscores the role of mobile elements as reservoirs allowing adaptability to changing environments. Our findings expand the Australian genomic catalogue beyond outbreak strains, reveal extensive accessory-genome variability in tropical waters and underscore the need for One-Health surveillance frameworks that monitor virulence and resistance markers beyond the canonical hemolysin genes.

## Graphical abstract

High genomic diversity of *Vibrio parahaemolyticus* from underexplored tropical northern Australia: genomic plasticity with pathogenic potential demands a One Health surveillance approach.

**Impact Statement**

With a warming climate, the marine bacterium *Vibrio parahaemolyticus* is increasingly responsible for foodborne outbreaks and skin and soft tissue infections. Despite its growing public health significance, little is known about its genomic diversity in Australia, apart from a select number of strains linked to a recent gastroenteritis outbreak caused by contaminated oysters. We address this gap by exploring the genomic diversity of *V. parahaemolyticus* isolates from environmental and clinical sources in the wet–dry tropics of northern Australia. We found a high genomic plasticity and distinct lack of spatial structure amongst the Australian genomes intermixing with Asian and South American isolates, reflecting the limited geographic structuring in the marine environment. A clinical gastrointestinal isolate contained the pathogenicity island VPaI-ß, including the haemolysin toxin gene *trh* with the toxin displaying an A187S change not present in the other TRH sequences of the isolates analysed in this study. Wound-associated strains lacked haemolysins, reinforcing the need for surveillance panels that extend beyond these genes to reflect the broader clinical and environmental diversity of *V. parahaemolyticus*. Notably, one wound strain carried a plasmid with a *Vibrio alginolyticus pilT* copy which is linked to twitching motility of the type IV pilus. Twitching motility is associated with wound infections, and it remains to be determined whether this extra *pilT* copy confers a selective advantage to colonize tissue. This study increases our understanding of the ecology of *V. parahaemolyticus*, emphasizing the need for One Health surveillance to monitor its emergence in diverse environments and manage associated risks.

## Data Summary

Sequence data generated by this study have been deposited in the Short Read Archive (BioProject accession number PRJNA1194145, SAMN45170680 to SAMN45170708). The authors confirm that all supporting data have been provided within the article or through supplementary data files.

## Introduction

Non-cholera *Vibrio* species, including *Vibrio parahaemolyticus*, occur naturally in estuarine environments where their prevalence is shaped primarily by sea surface temperature, salinity and nutrients [1]. Their geographical range is expanding with climate change [2,4], leading to more frequent gastroenteritisoutbreaks [5,8]. While accurate *Vibrio* speciation is crucial for public health monitoring, detecting virulence genes in non-cholera *Vibrio* species presents challenges due to their large genetic diversity [9,11] and an arsenal of proteins contributing to their pathogenicity [12,14]. The main toxins of *V. parahaemolyticus* causing gastroenteritis are thermostable direct haemolysin (TDH) and TDH-related haemolysin (TRH) encoded by *tdh* and *trh* genes [15]. Whole-genome sequencing (WGS) has revolutionized our knowledge of non-cholera *Vibrio* species [16,18] and has shed light on environmental adaptation and evolution of virulence and antibiotic resistance, the latter of which is a growing concern [2,19, 20].

In Australia, *V. parahaemolyticus* is linked to increasing gastroenteritis outbreaks from consumption of raw seafood, in particular oysters [21,22], a significant commodity in Australia [23,24]. In the wet–dry tropics of northern Australia, gastroenteritis caused by *V. parahaemolyticus* is a notifiable disease with a 5-year mean of annual 1.8 *Vibrio* food poisoning cases [25]; 20 superficial and deep tissue infections were recorded between 2015 and 2022 [26] and a fatal necrotizing fasciitis case in 2001 [27]. Limited WGS data are publicly available for Australian *V. parahaemolyticus* isolates, and most are clinical and outbreak related, with none from northern Australia. We selected 29 locally acquired *V. parahaemolyticus* isolates for WGS, prioritizing those from (1) urban and remote locations, (2) a range of animal hosts and (3) human clinical samples. Our objectives were to (a) conduct a phylogenetic analysis of these isolates compared to other Australian and global *V. parahaemolyticus* genomes while accounting for homologous recombination, (b) screen these isolates for *Vibrio* virulence genes and antimicrobial resistance (AMR) markers in the accessory genomes and (c) compare the genomic islands of two isolates from northern Australia using hybrid assemblies of short and long sequencing reads.

## Methods

### *V. parahaemolyticus* collection and sample processing

**Fig. 1.**
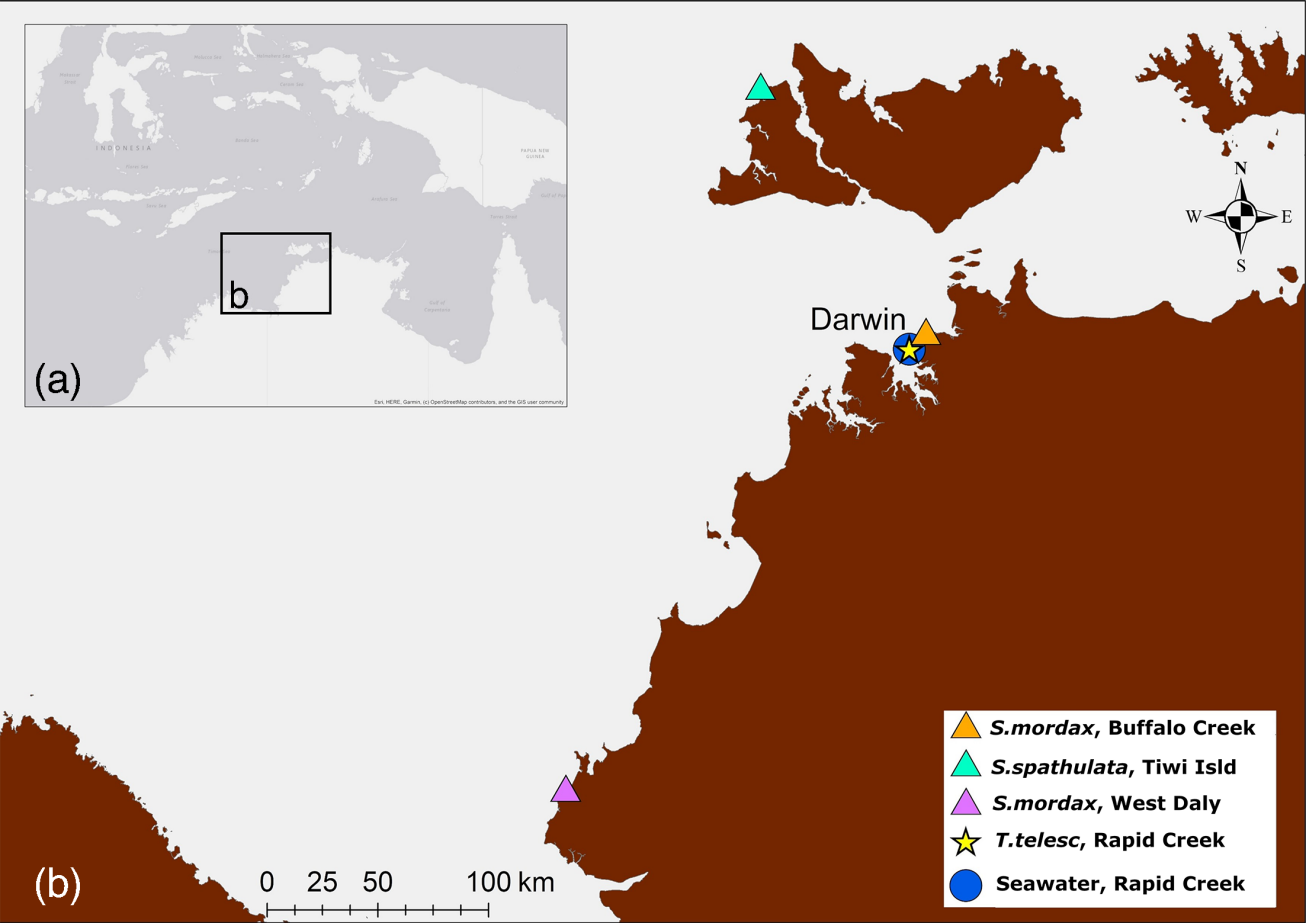
(a) Map of the maritime region (grey shaded) between northern Australia, Papua New Guinea and Southeast Asia; (**b**) inset showing the *V. parahaemolyticus* sample collection sites for M18-M118 in the southeast of the Timor Sea bordering northern Australia*.* Maps created in ArcGIS v10.4.1 (ESRI, Redlands, CA, USA).

Twenty-nine *V. parahaemolyticus* isolates were selected for WGS over 3 years (2020–2022) consisting of 5 isolates of human source (4 wound, 1 faecal), an aquaculture barramundi fish (*Lates calcarifer*) that died from an unverified cause, wild snails (8), wild oysters (11) and seawater (4) from 3 areas in the Western Top End of Australia (Fig. 1, Tables 1 and S1, full details in File S1, available in the online Supplementary Material).

**Table 1.** *V. parahaemolyticus* isolates sequenced in this study

Isolates	Collection location^1^	Collection source	Collection year	Virulence genes^2^	Antimicrobial Res genes^2^	Plasmid detected^3^	ST^4^	Same ST previously reported in (PubMLST, Sept 2024)
RDH1	N/A human^5^	Clin_Wound	2022		*fosA*		3,589	Novel ST
RDH2	N/A human	Clin_Wound	2022				3,587	Novel ST
RDH3	N/A human	Clin_Faecal	2022	*trh*, *hlyB*, *vtrB*		CP089144, CP097358.1	2,901	Clin, Australia (2022)
RDH4	N/A human	Clin_Wound	2022			NC_010113	3,585	Novel ST
RDH5	N/A human	Clin_Wound	2022			CP020079	2,014	Crust., Venezuela (2018)
M18	Rapid Creek	Seawater	2020				3,584	Novel ST
M19	Rapid Creek	Seawater	2020				3,590	Novel ST
M20	Rapid Creek	Seawater	2020				3,578	Novel ST
M21	Rapid Creek	Seawater	2020				3,580	Novel ST
M24, 26, 28–31	Rapid Creek	*T. telescopium* ^6^	2020				3,581	Novel ST
M25	Rapid Creek	*T. telescopium*	2020				2,249	Env, Thailand (2017)
M27	Rapid Creek	*T. telescopium*	2020				3,588	Novel ST
M35	West Daly	*S. mordax* LA^7^	2021				3,582	Novel ST
M36-38	West Daly	*S. mordax* LA	2021				3,586	Novel ST
M39	West Daly	*S. mordax* LA	2021				2,058	Env, France (2011), Spain (2021); clin, China (2019), USA (2023)
M40	West Daly	*S. mordax* LA	2021				3,576	Novel ST
M51	Tiwi Islands	*S. spathulata*	2021				3,574	Novel ST
M56	Tiwi Islands	*S. spathulata*	2021				3,579	Novel ST
M116	Buffalo Creek	*S. mordax* LA	2021				3,577	Novel ST
M117	Buffalo Creek	*S. mordax* LA	2021				3,575	Novel ST
M118	Buffalo Creek	*S. mordax* LA	2021				3,583	Novel ST
M132	Darwin region	Barramundi^8^	2022		*qnrS5*	CP007006	2,013	Shrimp, Malaysia (2018)

**Fig. 2.**
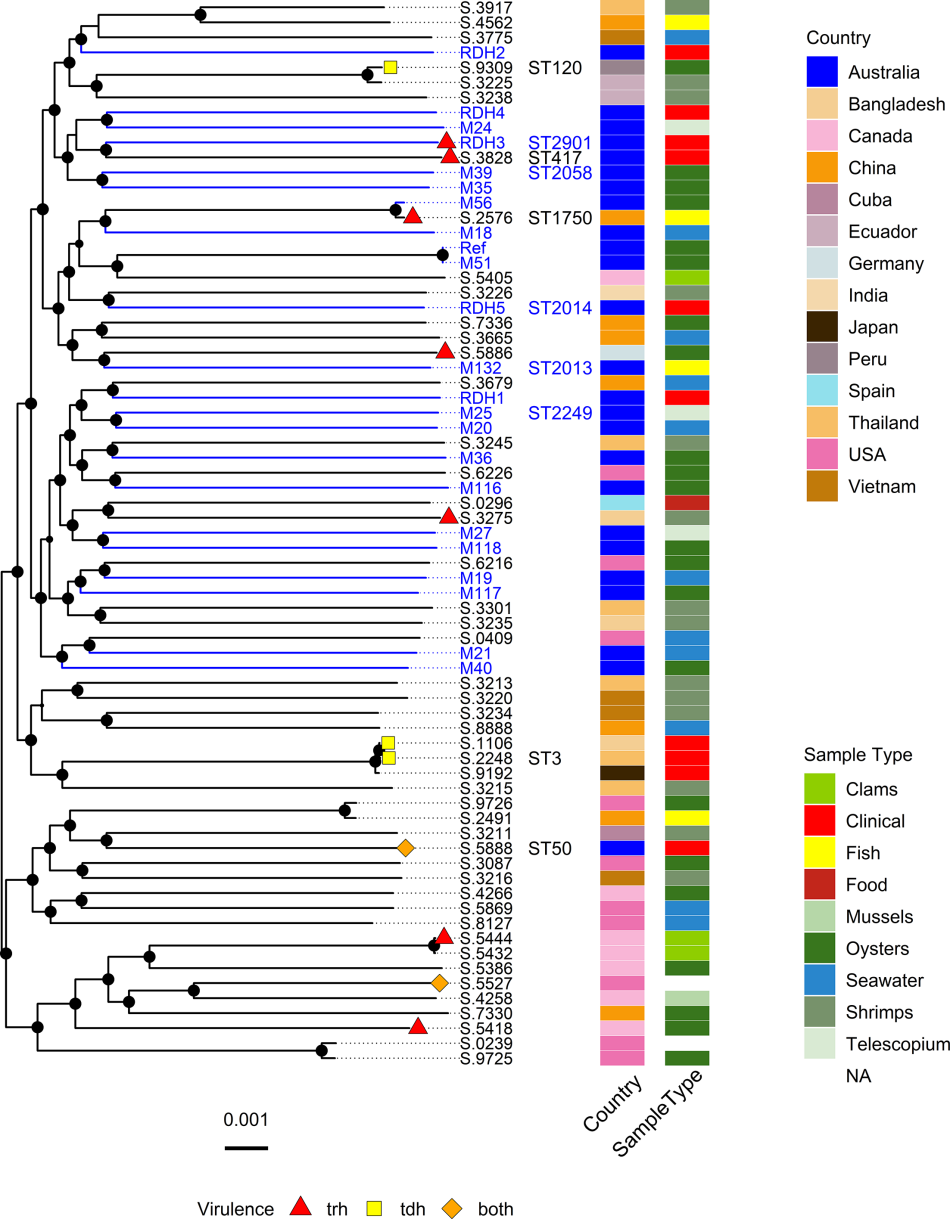
ML mid-rooted phylogenetic tree of 22 distinct *V. parahaemolyticus* isolates from this study (blue branches) and 48 public genomes. Recombinogenic sites were filtered. Bootstrap support for branches above 80% is shown with a dot. The scale bar indicates substitutions per site. Tip labels show the isolate names of this study and the last four digits of the SRA public genomes (Table S2). Select STs include, in blue, previously described STs found in this study, the Australian outbreak-related strains ST50 (SRR17035888) and ST417 (SRR17673828) [21], the pandemic strain ST3 (ERR5319192, SRR5071106 and SRR21252248) and outbreak strains ST120 (SRR2559309) [80] and ST1750 (ERR12422576) [64]*.* The presence of virulence genes *trh*, *tdh* or both is indicated*.*

1See also Fig. 1; 2,3see methods and Tables S3 and S4 for more details; 3based on MOBsuite with the exception of RDH3; 4MLST sequence type (ST); 5human clinical isolates from Territory Pathology with no patient information available; 6*T. telesc*, *Telescopium telescopium*; 7*S. mordax* or *spathulata Saccostrea mordax* or *spathulata* – LA lineage A; 8 captive fish – *L. calcarifer*.

Whole tissue samples of molluscs and barramundi heart tissue were homogenized and spread onto CHROMagar^™^ Vibrio (CAV) plates for overnight incubation at 30 °C. Seawater was filtered (200–500 ml) onto 0.45 µm membranes which were placed on CAV plates. The identity of individual mauve colonies was determined by qPCR targeting the *V. parahaemolyticus tlh* gene [28]. Five clinical *V. parahaemolyticus* isolates were obtained as cultures on HBA plates from Territory Pathology at Royal Darwin Hospital.

### Antimicrobial susceptibility testing using E-tests

Susceptibility testing for fluoroquinolones (ciprofloxacin and norfloxacin) and fosfomycin was conducted on RDH1 (wound) and M132 (barramundi) after *in silico* genomic screening predicted resistance to quinolones (M132) and fosfomycin (RDH1). Isolates RDH3, *Escherichia coli* ATCC15766 and *Pseudomonas aeruginosa* ATCC9027 were included as controls. The bacteria were grown on Mueller–Hinton agar, and MICs were determined using E-tests (bioMérieux).

### Isolation of DNA from *V. parahaemolyticus*

Glycerol stocks of 29 *V*. *parahaemolyticus* strains stored at −80 °C were streaked onto marine agar and incubated overnight (clinical isolates 35 °C, environmental 30 °C). A single colony was selected to inoculate 5 ml of marine broth. DNA was extracted from 1.5 ml of overnight culture using the GenElute^™^ Bacterial Genomic DNA Kit (Sigma-Aldrich, MO, USA).

### PCR screening of species-specific genes and virulence markers

qPCR testing for the *V. parahaemolyticus*-specific gene *tlh* and virulence genes *trh* or *tdh* was conducted on all isolates as described in Nordstrom *et al*. [28].

### Whole-genome sequencing

WGS was performed at the Australian Genome Research Facility on the Illumina NovaSeq 6000 (150 bp PE). Long reads were also obtained for two isolates (rock oyster M51 and faecal isolate RDH3) (File S1). All WGS files have been deposited under BioProject accession number PRJNA1194145.

### Bioinformatic WGS data analysis

See File S1 for full details. Illumina short-read sequencing data were checked using FastQC [29], and sequence coverage was examined in Tablet (https://ics.hutton.ac.uk/tablet/) with BAM files generated in SPANDx v4.03 (https://github.com/dsarov/SPANDx) [30].

#### Phylogenetic and recombination analysis

A core genome alignment was conducted on the 29 *V*. *parahaemolyticus* genomes from this study (see Table 1) using the default settings of Snippy v4.6.0 (https://github.com/tseemann/snippy) and the hybrid assembly of M51 as reference genome (2 contigs reflecting 2 chromosomes). The final phylogeny represented 22 distinct core genomes of this study (7 core genomes were excluded as identical to other isolates of the same samples) (see Table 1) and 48 select public global *V. parahaemolyticus* genomes of outbreak-associated (no clonal redundancy) and environmental strains (Table S2). Using ClonalFrameML v1.12, homologous recombinogenic sites were assessed [31] and filtered with cfml-maskrc (https://github.com/kwongj/cfml-maskrc). A maximum-likelihood (ML) tree was generated in IQ-TREE v2.2.0.3 [32] using bootstrapping (1,000 replicates) [33] and the nucleotide substitution model TVM+F+I+I+R9 selected by the ModelFinder and BIC. This was based on 93% constant sites and 343,386 variant sites. The tree was visualized using R packages ggtree [34], phytools [35], ape [36] and cowplot.

#### Genome assembly

Genomes were assembled *de novo* using Shovill 1.1.0 (https://github.com/tseemann/shovill). Assembly quality was assessed with QUAST (https://github.com/ablab/quast). The number of contigs varied between 33 and 85, and N50 was between 195,860 and 680,144 bp. All assemblies were screened for non-*V. parahaemolyticus* DNA using Kraken-2 [37].

#### MLST molecular typing

MLSTs were assigned *in silico* using the MLST assignment tool ‘mlst’ (Seemann, T, https://github.com/tseemann/mlst) based on the *V. parahaemolyticus* MLST scheme and uploaded to the PubMLST website (https://pubmlst.org/) [38,39].

#### Virulome

The assemblies were screened for virulence genes using ABRicate (Seemann, T, https://github.com/tseemann/abricate) with the VFDB database and default minimum 80% identity [40]. Further, *toxR*, *toxS* (*V. parahaemolyticus* strain U-5474, GenBank AB029915.1 and .2) and *vtrB* (*V. parahaemolyticus* strain MAVP-RPI, MF066647.2: bp 65052–65585; locus tag MAVP-RPIeRC_00057) were screened using SRST2 v0.2.0 (https://github.com/katholt/srst2/tree/master) [41].

A MAFFT sequence alignment (v 7.526) [42] was conducted on the amino acid sequence of 12 TRH-1/-2 variants with 9 from the 77 *V*. *parahaemolyticus* genomes examined in this study and 3 reference TRH variants [TRHx reference of VFDB (Genbank AAB29385), UniProt A0A162SI74 TRH1 variant and UniProt A0A162SI74 TRH2 variant]. The amino acid sequence of the latter was identical to RDH3 and its AlphaFold-predicted protein structure (AF-A0A162SI74-F1-v4.pdb) used to predict the structure of RDH3-TRH2 in Jalview 2.11.4.1 [43,44].

#### Resistome

The assemblies were screened for acquired antimicrobial resistance genes (ARGs) using ABRicate (Seemann, T, https://github.com/tseemann/abricate) which uses the NCBI database AMRFinderPlus [45].

#### Plasmid detection

Assemblies were screened for the presence of plasmids using MOBsuite [46]. A blast search was conducted for the RDH3 small plasmid.

#### Genome mapping of genomic islands for RDH3 and M51

The hybrid assembler Unicycler (https://github.com/rrwick/Unicycler) [47] on Galaxy Australia (https://usegalaxy.org.au) [48] was used for two genomes (RDH3 and M51) with short-read and long-read data available. Prokka [49] was used to annotate the genomes which were uploaded to IslandViewer4 (https://www.pathogenomics.sfu.ca/islandviewer/) to map genomic islands (reference genome *V. parahaemolyticus* RIMD 2210633) [50]. Chromosome 2 of RDH3 and M51 was aligned and visualized with AliTV v1.0.6 [51] and LASTZ as the pairwise sequence aligner. Annotated genes of the pathogenicity island VPaI-β were mapped using Proksee [52].

#### Pangenome analysis

A pangenome of the 77 *V*. *parahaemolyticus* isolates (29 from this study and 48 global isolates) was constructed in Roary [53] and visualized in Phandango after annotating all assembled genomes with Prokka.

## Results

### Isolate diversity and molecular typing

The 29 *V*. *parahaemolyticus* isolates of environmental and clinical origin from the wet–dry tropics of northern Australia displayed large genomic diversity with 22 different sequence types (STs). The majority of *V. parahaemolyticus* isolates (83%, 24 out of 29) had a novel ST including 11 novel MLST alleles (Table 1). There were no STs shared between isolates from different locations and sources. Six isolates from pooled *T. telescopium* collected at the same time and location shared the same ST and so did three isolates from pooled *S. mordax*. Five of the 22 STs were already listed in PubMLST (Table 1). This included ST2901 (faecal human sample RDH3 from 2022) which was also detected in a clinical sample from South Australia from the same year. ST2058 isolated from an *S. mordax* in the remote West Daly region has previously been detected in two clinical cases in China (2019) and the USA (2023), while ST2013 isolated from a sick captive fish has been described in Malaysia (2018) in shrimp with acute hepatopancreatic necrosis disease [54].

### Core genome phylogenetic analysis

Core genome phylogenetic analysis showed high diversity with long terminal branches for most genomes and a lack of clear clustering (Fig. 2). The Australian isolates were dispersed amongst isolates from Asia and South America. One Australian clinical isolate (ST50), related to a 2021 food poisoning outbreak [21], resided in a cluster with mainly North American isolates.

Virulence genes *tdh*, *trh*, *hlyB* and *vtrB* were spread across the tree reflecting their presence on pathogenicity islands independent of the core genome. M56 (ST3579) isolated from oysters at Tiwi Islands shared a cluster with shorter branches with *trh*-positive ERR12422576 (ST1750) from a fish in China with 5,851 core SNPs between them and 6 of 7 MLST alleles shared.

### Recombination analysis

There was a recombination-to-mutation rate (R/theta) of 0.67, indicating that for every one recombination event, there were 1.5 mutation events. A comparison of r/m (the ratio of the number of SNPs introduced by recombination over those introduced by mutation) showed that for 74% of isolates and 90.0% (125 out of 139) of nodes, the genetic divergence from the reference M51 core genome was mainly driven by vertically inherited mutations with an r/m ratio of 0.04–0.42. For 13 isolates (and one node), the r/m ratio was large with 4–27, suggesting that their genetic divergence from M51 was driven by recombination. This included the oyster isolate M56 and its single-locus variant ST1750 recovered from a fish in China (ERR12422576). Filtering of recombinogenic regions resulted in rearrangements in the broad cluster containing Australian, Asian and South American isolates reflecting their short internal branch lengths, while the topology of the North American cluster remained largely the same pre- and post-filtering (tanglegram in Fig. S2, File S1).

### Pangenome

A pangenome analysis of the 77 *V*. *parahaemolyticus* genomes revealed 23,377 different annotated genes of which 85.3% of genes were defined as accessory genome occurring in <99% of genomes (Fig. S3). A hierarchical tree showed the clustering of five of nine genomes which were *trh* and *vtrB* positive. The gastrointestinal strain RDH3 and Australian outbreak-related strains SRR17035888 and SRR17673828 [21] were also part of this cluster. These genomes also contained other genes associated with pathogenicity islands, including the urease gene cluster, genes encoding the nickel import system and T3SS-associated genes (*hrcN* and *yscU-2*). M24, 26, 28–31 were collected from snails at the same time and location and shared the same core genome, but M26 lacked a set of accessory genes associated with stress response and potential virulence factors (Fig. S3 and File S1).

### Virulome and TRH variants

All isolates from this study were PCR positive for the *V. parahaemolyticus* species marker gene *tlh* and negative for *tdh*. The faecal clinical isolate, RDH3, was PCR positive for *trh*. For the majority of virulence markers, the isolates were either all positive or negative with no variation across isolates (Table S3). Exceptions were *trh (trhx*), the alpha haemolysin operon gene *hlyB* and *vtrB*, which encodes a ToxR-like transcriptional regulatory protein controlling the expression of pathogenicity island genes [55]. These were detected in RDH3. The detection of *vtrB* showed 100% coverage (534 bp) and 98.7% identity. The identity of *trh* was lower with 86% and also only 82% for *hlyB* (100% coverage for both with 570 bp and 2,123 bp) (File S1). The *tdh* gene was detected in 5 and *trh* in 8 of the 48 public isolates (with 84–85% identity for 6 and 99–100% for 2 isolates) (Fig. S3).

#### TRH variants and characteristics

Two main *trh* variants have been described in *V. parahaemolyticus*, with *trh1* and *trh2* sharing 84% sequence identity [56]. *Trh2* was the variant found in RDH3. The amino acid sequence (189 amino acids) was identical to UniProt TRH2 AOA162S174 from a US clinical strain 4591 [44]. The predicted protein structure of RDH3 TRH2 showed ten beta-strands and one alpha-helix between the seventh and eighth strand (Fig. S4A). There were two TRH1 sequences in this study, of which one was from the Australian clinical isolate SRR17035888. Seven TRH sequences belonged to the TRH2 group with 89–100% amino acid sequence identity amongst them and 84–87% identity to TRH1 (Fig. S4B). Mutations unique to RDH3-TRH2 (and UniProt AOA162S174) included a hydrophobic alanine instead of hydrophilic serine for all other TRH sequences in this study (A187S near the C-terminus of the protein). See File S1 for full details.

### Genomic mapping and islands of clinical RDH3 and environmental M51

**Fig. 3.**
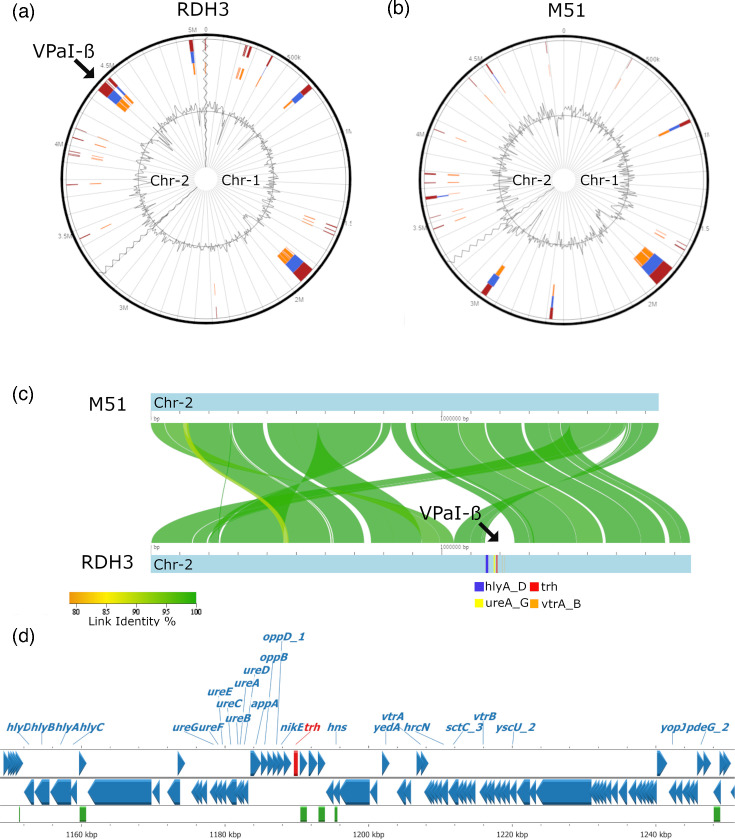
Genomic island predictions for (a) RDH3 and (b) M51 using IslandViewer4 and the *V. parahaemolyticus* genome RIMD 2210633 as reference. Chromosome 1 and 2 boundaries are marked by the wavy line. The inner ring shows the % GC skew, and the orange-blue-red sections mark the predicted genomic islands based on SIGI-HMM, IslandPath-DIMOB and the integrated results. The arrow in (a) shows the pathogenicity island VPaI-β on RDH3 chromosome 2 with the mapped virulence genes *hlyB*, *trh* and *vtrB*. (**c**) Chromosome 2 genome alignment of M51 and RDH3 showing several genome rearrangements. The arrow marks the VPaI-β island with genes *hylA* to D, *ureA* to G, *trh* and *vtrA* and *B*. (**d**) Mapped genes of VPaI-β. Blue triangles show predicted coding regions (*trh* is in red) – labels for genes encoding hypothetical proteins are not shown. Green areas mark predicted mobile genetic elements (*tnpA*, transposase genes) based on mobileOG-db [81].

Hybrid assembly of RDH3 resulted in three contigs corresponding to two chromosomes of 3.21 and 1.86 Mb and a small plasmid of 3.8 kb (Fig. S5). M51 had two contigs of 3.25 and 1.75 Mb with no plasmid. Genomic island predictions revealed a 99–118 kb island on chromosome I in the same region (1.8–1.9 Mb) for both isolates (Fig. 3a). The island contained predicted transposases, toxin–antitoxin modules and various stress-response genes (File S1). Many of these genes were ubiquitous across tested isolates, suggesting this to be a common fitness island.

RDH3 had a second 102 kb genomic island on chromosome II which was absent in M51 (Fig. 3a–c). This island encoded the three detected virulence genes (*hlyB*, *trh* and *vtrB*), as well as a urease gene cluster, nickel import system and some type III secretion system-related genes (Fig. 3d). This island is known as pathogenicity island *trh*PAI [57] or VPaI-β [58].

### Plasmid detection

A plasmid was detected in 4 of the 29 isolates. Three of these were from clinical isolates (RDH3-5) and one from a diseased fish (M132) (Table 1).

The small plasmid (3.8 kb) in RDH3 was not detected by MOBsuite but was apparent in the hybrid assembly and on an agarose gel of the genomic DNA. A blast search revealed a 92% identity to the plasmid pSO5Y of a *Vibrio cholerae* strain for 55% of its length, while for another 45%, it showed 96% identity to the 1.8 kb plasmid pVP-16-VB00198-2 of the *V. parahaemolyticus* strain 16-VB00198 (Germany, oyster, 2022). Genome annotation predicted a cold shock-like protein CspG gene in the latter region.

A contig (79.5 kb) of the RDH4 isolate had high similarity [mash distance 0.022, average nucleotide identity (ANI) ~97.5%] to a *Vibrio* spp. plasmid p0908 (NC_010113) (81.4 kb, USA, sediment, 1998) [59]. RDH5 had a contig (170.4 kb) with ANI 98.8% to a *Vibrio campbellii* plasmid pVCGX2 (204 kb). Gene annotation revealed a *pilT* on the RDH5 plasmid which encodes the ATPase component of the type IV pilus – also called twitching motility protein. This amino acid sequence differed from the core genome-encoded PilT-1 and -2, and a BLASTp search showed that it was identical to a PilT encoded by a plasmid in *Vibrio alginolyticus*.

Finally, two contigs (combined length, 86.5 kb) of M132 showed very high similarity to the *V. parahaemolyticus* plasmid pVPUCMV (ANI 99.1%) (88.5 kb, sediment, Spain, 2002) [60]. The M132 plasmid carried the *pulD (gspD*) gene, encoding the T2SS secretin component, but lacked other T2SS genes required to form a functional secretion system (File S1).

### AMR and E-test results

*In silico* screening revealed the presence of beta-lactamase *blaCARB* gene variants and the *tet(34,35)* genes in all isolates (Table S4). The genome of M132 (aquaculture fish) also had the *qnrS5* gene (NG_050546.1) encoding the quinolone resistance pentapeptide repeat protein. The FosG/FosC2 family fosfomycin resistance glutathione transferase gene (NG_050560.1) was found in the clinical isolate RDH1. *In silico* screening of the public genomes in this study revealed one more *V. parahaemolyticus* isolate (SRR19513301, Thailand, shrimp) with the fosfomycin resistance gene and one isolate (ERR12422576) with the *qnrS5* gene. Similar to M132, ERR12422576 was also isolated from a fish (in China).

E-test results showed that all three tested *Vibrio* isolates (RDH1, RDH3 and M132) were susceptible to the two tested fluoroquinolones with MICs 0.13–0.19 mg l^−1^ (0.13–2.0 mg l^−1^ for controls *E. coli* and *P. aeruginosa*). While EUCAST susceptibility breakpoints are not defined for fosfomycin and *Vibrio* spp., we still conducted the testing and confirmed a reduced susceptibility of RDH1 to fosfomycin with an MIC of 24 mg l^−1^ which compared to 3 mg l^−1^ for M132 (0.75–3 mg l^−1^ for *E. coli* and *P. aeruginosa*). RDH3 also showed a reduced susceptibility to fosfomycin with an MIC of 32 mg l^−1^.

## Discussion

We found that the *V. parahaemolyticus* genomes from the wet–dry tropics of northern Australia intermixed with Asian and South American isolates within the broad VppAsia cluster [61] consistent with its wide geographic distribution and dispersal via ocean currents. Some strains from this study shared the same STs as environmental isolates from Europe and the USA where long-range dispersal might be facilitated by human activity such as shipping, aquaculture trade or microplastics [61,63].

High recombination rates contribute to the genomic diversity of *V. parahaemolyticus* [61,64, 65]. We found a high recombination-to-mutation signal in a cluster comprising the Australian oyster isolate M56 and its single-locus variant ST1750, a *trh*-positive fish isolate from China, consistent with previous reports of horizontal gene transfer in ST1750 [64]. Further evidence of genomic plasticity came from co-collected snail isolates that shared the same core genome but differed in accessory genes, highlighting the species’ capacity to readily acquire or lose DNA.

The gastroenteritis isolate RDH3 was recovered during a 2021–2022 Australian food poisoning outbreak (268 reported cases) linked to contaminated South Australian oysters [21,25] but differed from the outbreak strains (ST50 and ST417), carrying ST2901 suggesting that it was not part of the outbreak. RDH3 contained the *trh2*-positive pathogenicity island VPaI-β [58]. Of the two main *trh* variants, *trh1* generally co-occurs with *tdh* on pathogenicity island vPaI-7, whereas *trh2* resides on the VPaI-β island – the configuration seen in RDH3. Its TRH2 sequence matched the type-V variant [66] (for residues 93–142) associated with Asian strains. RDH3 TRH2 carried several non-conservative substitutions including an A187S change near the C-terminus, a region implicated in tetramer formation [67]. Mutagenesis studies would be needed to determine the functional impact of these substitutions. Ultimately, *trh* sequence variation complicates PCR assay design, with the adjacent *ureR* gene suggested as a more conserved surrogate target [68].

The wound isolates lacked haemolysin genes and were from polymicrobial infections, making it uncertain whether *V. parahaemolyticus* was the primary pathogen. Nonetheless, severe wound infections due to *V. parahaemolyticus* are well documented in northern Australia [26,27, 69], and the species’ intrinsic virulome of adherence, pili and effector systems [70] may suffice to colonize wounds. Two wound isolates (RDH4 and RDH5) carried plasmids, one encoding a twitching motility-associated *pilT* variant described in *V. alginolyticus*; twitching motility has been linked to wound colonization [71,72]. Whether this additional *pilT* conferred advantage beyond the genome-encoded copies is unclear. A small plasmid was also detected in the gastrointestinal isolate RDH3, which appeared to be a chimaera: half resembling a plasmid which had been described in *V. cholerae* and half a *V. parahaemolyticus* plasmid. Natural plasmid chimaeras have been reported in *Pseudomonas* [73], but to our knowledge, not previously in *Vibrio*.

The diseased aquaculture fish carried strain ST2013, which was previously associated with acute hepatopancreatic necrosis disease (AHPND) in shrimp aquaculture [73]. AHPND is caused by the Pir toxin encoded on the pVA plasmid [54]. M132 (ST2013) was the only environmental isolate in this study with a plasmid; although unrelated to pVA and lacking *pir* genes, it illustrates that ST2013, like other *V. parahaemolyticus* strains, can harbour diverse plasmids that may confer fitness benefits under conditions such as high-density aquaculture.

The clinical wound isolate RDH1 contained the FosG/FosC2 family fosfomycin resistance gene with reduced susceptibility to fosfomycin. Fosfomycin is not part of the standard drug treatment for *Vibrio* infections in humans but is used in aquaculture in some countries [74]. It is also an old antibiotic experiencing a revival for the treatment of urinary tract infections, particularly those caused by *E. coli*. Fosfomycin resistance is increasingly reported in *E. coli* (including in wastewater) and aquaculture *Vibrio* [74,77], highlighting aquatic environments as reservoirs of ARGs with potential for horizontal transfer [78,79].

Data on *V. parahaemolyticus* in Australia have historically been limited, as illnesses caused by *V. parahaemolyticus* have not been nationally notifiable [22]. This, coupled with a lack of environmental isolates, has hindered in-depth analysis of *V. parahaemolyticus* ecology in Australia and the potential risks it may pose to aquaculture and public health. In this work, genomic data of *V. parahaemolyticus* from coastal regions in northern Australia were analysed for the first time. Some of these areas were remote and difficult to reach, and accordingly, a limitation of this study was that the sample numbers and locations were limited.

## Conclusions

We explored the genomic composition of *V. parahaemolyticus* strains from the wet–dry tropics of northern Australia, extending the publicly available genomic data for *V. parahaemolyticus* beyond food poisoning outbreak-related strains from southern Australia. With chronic diseases increasing, this study provided insight into strains lacking haemolysin genes but associated with wound infections. In a warming climate, understanding the ecology of *V. parahaemolyticus* alongside expanded genomics will aid surveillance strategies that move beyond haemolysin markers to capture regional virulence and AMR gene pools. These findings reinforce a One Health perspective, linking environmental reservoirs, aquaculture and human infections to better inform public health measures.

## Supplementary material

10.1099/mgen.0.001536 Uncited Fig. S1.

## Abbreviations

AHPND: acute hepatopancreatic necrosis disease
AMR: antimicrobial resistance
ANI: average nucleotide identity
ARG: antimicrobial resistance gene
CAV: CHROMagar^™^ Vibrio
ML: maximum likelihood
ST: sequence type
TDH: thermostable direct haemolysin
TRH: TDH-related haemolysin
WGS: whole-genome sequencing
