# Supplementary material for: High genomic diversity of Vibrio parahaemolyticus from underexplored tropical northern Australia: a baseline for future surveillance
Source: Microb Genom. 2025 Oct 27;11(10):001536. doi: 10.1099/mgen.0.001536 (PMC12558636; doi:10.1099/mgen.0.001536)
Supplement: Uncited Fig. S1. [file mgen-11-01536-s001.pdf]

Supplementary Material to MGEN-D-25-00019 "High genomic diversity of* Vibrio parahaemolyticus *from underexplored tropical northern Australia: a baseline for future surveillance"

Contents

File S1 2

Supplementary Methods 2

Supplementary Results 7

Supplementary Tables 13

Supplementary Table 1 13

Supplementary Table 2 16

Supplementary Table 3 18

Supplementary Table 4 20

Supplementary Figures 22

Supplementary Figure 1 22

Supplementary Figure 2 24

Supplementary Figure 3 25

Supplementary Figure 4 27

Supplementary Figure 5 31

References 32

File S1

Supplementary Methods

Source of V. parahaemolyticus

Twenty-nine *Vibrio parahaemolyticus* isolates were selected for whole genome sequencing over three years (2020-2022) with isolates of human source (n=5, four wound and one faecal), an aquaculture barramundi (*Lates calcarifer*) that was dying from an unverified cause, wild snail (8), wild oysters (11) and seawater (4) from three areas in the Western Top End of Australia (Figure 1, Table 1, Supplementary Table 1, more details in file S1). Excluding clinical and aquaculture isolates, these were Darwin (15), and remote locations near Tiwi Islands (2) and the West Daly region (6) (Figure 1). Specifically, *V. parahaemolyticus* was isolated from a wild marine gastropod snail *Telescopium telescopium* (8 isolates) in Rapid Creek which is an urban tidal creek in Darwin. This snail is commonly eaten by Indigenous people living in and around Darwin, both cooked and raw. Four isolates were from Rapid Creek water collected at the same time as the snails. *V. parahaemolyticus* was also isolated from a wild tropical rock oyster species (S*accostrea mordax* / lineage A) from the West Daly region (six isolates) and an urban Buffalo creek impacted by treated sewage effluent (three isolates). Isolates were also sourced from a wild tropical rock oyster species (*Saccostrea spathulata*) collected from the Tiwi Islands (two isolates). The oysters from the remote locations were collected as part of an Australian Fisheries Research and Development Corporation project (FRDC project 2018-005) and molluscs from Darwin were collected under Special Permit No 2020-2021/S17/3496 from the Northern Territory Government. We also isolated *V. parahaemolyticus* from a captive barramundi (*Lates calcarifer*) (M132) that was dying from an unverified cause, and a heart tissue sample was provided to us via an attending veterinarian. We obtained five human clinical *V. parahaemolyticus* isolates (RDH1-5) from five patients by Territory Pathology, namely one faeces isolate which was from a patient with gastroenteritis (RDH3) and four from wound infections some of which were polymicrobial (RDH1-2,4-5) (Supplementary Table 1). No further clinical nor travel information were available for the human isolates.

Source (host) processing and V. parahaemolyticus *isolation*

Molluscs (snail and oysters) were scrubbed under running potable water and opened aseptically. The whole tissue was homogenized using an Ultra-Turrax IKA T18 (IKA Works, Malaysia) with 1x PBS and 100 mL of the homogenate spread onto CHROMagar^{TM} Vibrio (CAV) plates and incubated overnight at 30°C. The barramundi heart sample was dissected aseptically, and homogenized heart tissue was spread onto CAV plates as described above. *V. parahaemolyticus* was isolated from seawater by filtering 200-500 mL onto 0.45 µm membranes and placing the membranes on CAV plates for overnight incubation. The identity of individual mauve colonies was determined by qPCR targeting the *V. parahaemolyticus tlh* gene [1] and further confirmed by BLAST analysis

(https://BLAST.ncbi.nlm.nih.gov/BLAST.cgi) of the sequenced *hsp60* gene [2]. Five clinical *V. parahaemolyticus* isolates were obtained as cultures on HBA plates from Territory Pathology at Royal Darwin Hospital.

Whole genome sequencing

Whole genome sequencing (WGS) were performed at the Australian Genome Research Facility (AGRF). All isolates were sequenced on the Illumina NovaSeq 6000, S1 flow cell (150bp PE). PrepM library processing was according to the default Illumina protocol. In addition, long-reads were also obtained for two isolates, namely M51 and RDH3, reflecting an environmental Northern Australia isolate (blacklip rock oyster, Tiwi Islands) and a faecal isolate. These were sequenced with the PacBio Sequel II instrument, on a SMRTcell 8M. PacBio libraries were prepared following the PacBio protocol "Procedure & Checklist – Preparing Multiplexed Microbial Libraries Using SMRTbell Express Template Prep Kit 2.0". The sequencing files have been submitted to the NCBI Sequence Read Archive under the BioProject accession number PRJNA1194145.

Bioinformatic WGS data analysis

Illumina short-read sequencing data was checked using FastQC [3] and sequence coverage examined in Tablet (https://ics.hutton.ac.uk/tablet/) using bam files generated in SPANDx v4.03 (https://github.com/dsarov/SPANDx)[4] and with the reference closed genome of *V. parahaemolyticus* RIMD 2210633 (NC_004603.1 and NC_004605.1). Sequence coverage was consistent with an average coverage depth typically above 250 reads for both chromosomes.

Phylogenetic and recombination analysis

A core genome alignment was conducted on the 29 *V. parahaemolyticus* genomes from this study (see Table 1) using the default settings of Snippy v4.6.0 (https://github.com/tseemann/snippy) and the long-read assembly of M51 as reference genome (2 contigs reflecting the 2 chromosomes). This locally collected strain was chosen as a reference to maximize the core alignment accuracy. Seven core genomes were identical to other isolates of the same samples (see Table 1) and were therefore excluded from the final phylogeny which represented one distinct core genome or isolate per sample (22 genomes) from this study and 48 select publicly available, global *V. parahaemolyticus* genomes (Supplementary Table 2). To prevent clonal redundancy amongst the public dataset i.e. clusters of near-identical outbreak-related isolates, we curated the set to include a balanced mix of outbreak-associated and environmental strains from different sources and countries.

The core alignment consisted of 90% constant sites and 486,402 variant sites. A maximum likelihood (ML) phylogenetic tree was generated in IQ-TREE v2.2.0.3 [5] using the nucleotide substitution model TVM+F+I+I+R10 selected by the ModelFinder and lowest BIC score [6]. Bootstrapping was performed using 1,000 replicates [7]. In the absence of a clear outgroup,

the tree was mid-rooted. Initial trials included a genome of the close neighbour *V. diabolicus* (SRR24070350) as outgroup, but this genome proved too divergent.

Using ClonalFrameML v1.12, homologous recombinogenic sites in the core genome were assessed and mean DNA import length, divergence of imported DNA (nu) and overall recombination to mutation rate (R/theta) computed [8]. Recombinogenic sites were filtered from the core alignment using cfml-maskrc (https://github.com/kwongj/cfml-maskrc) and a ML tree again generated in IQTree using bootstrapping and the nucleotide substitution model TVM+F+I+I+R9 selected by the ModelFinder. This was based on 93% constant sites and 343,386 variant sites. The tree was visualized using R packages ggtree [9], phytools [10], ape [11] and cowplot.

Unfiltered and filtered trees were compared in R (v4.3.1) with the help of a tanglegram and the packages ape [11] and dendextend [12]. Recombination-prone regions in the core genome were also assessed using Gubbins 3.3.1 [13] and recombination to mutation (r/m) ratios calculated. Core genome recombinogenic sites were visualized in Phandango v0.9 [14] including a ML tree generated with Gubbins and default RAxML v 8.2.12 and a Gamma GTR model [15].

Genome assembly

Genomes were assembled *de novo* using Shovill 1.1.0 which is based on SPAdes, with minimum length of 1,000 bp and default settings to correct sequencing errors and adaptors and limiting sequence coverage to 150 reads (https://github.com/tseemann/shovill). Assembly quality was assessed using QUAST (https://github.com/ablab/quast). The number of contigs varied between 33 and 85, and N50 was between 195,860 and 680,144 bp. All assemblies were screened for non-*V. parahaemolyticus* DNA using Kraken-2 [16]. Reads were assigned to *V. parahaemolyticus* at 80-92% for 20 genomes, 70-79% for eight including RDH1 and 64% for M117. The remainder mapped to closely related *Vibrio* species, including *V. vulnificus, V. harveyi, V. campbellii* or *V. alginolyticus*. Notably, the latter two also appeared in RDH1 originating from a wound where these species are unlikely to occur. Given that these closely related *Vibrio* species share many genomic segments, making accurate species-level classification challenging with Kraken2's kmer-based approach [17], we considered the non-*V. parahaemolyticus* reads more likely to be classifier artefacts than true contamination.

MLST molecular typing

Multi-locus sequence types (MLST) were assigned *in silico* using the MLST assignment tool “mlst” (Seemann, T, https://github.com/tseemann/mlst) based on the *V. parahaemolyticus* MLST scheme and uploaded to the PubMLST website (https://pubmlst.org/)[18, 19].

Virulome

The assemblies were screened for virulence genes using ABRicate (Seemann, T, https://github.com/tseemann/abricate) with the VFDB database and default minimum 80% identity (i.e. 80% identical nucleotides) [20]. A further three sequences of virulence candidates not listed in VFDB were screened using SRST2 v0.2.0 (https://github.com/katholt/srst2/tree/master) [21]. These were *toxR*, *toxS* (*V. parahaemolyticus* strain U-5474, GenBank AB029915.1 and .2), and *vtrB* (*V. parahaemolyticus* strain MAVP-RPI, MF066647.2: bp 65052-65585; locus tag MAVP-RPIeRC_00057).

A MAFFT multiple sequence alignment (v 7.526, FFT-NS-2 settings)[22] was conducted on the amino acid sequence of twelve TRH-1/-2 variants with nine identified amongst the 77 *V. parahaemolyticus* genomes examined in this study (using gene annotations based on Prokka – see section 2.6.8) as well as three reference TRH variants, namely the TRHx reference sequence of the VFDB database (Genbank AAB29385), a UniProt A0A162SI74 TRH1 variant, and a UniProt A0A162SI74 TRH2 variant. The amino acid sequence of the latter was identical to RDH3 and its AlphaFold predicted protein structure (AF-A0A162SI74-F1-v4.pdb) used to predict the structure of RDH3-TRH2 in Jalview 2.11.4.1 using Jmol [23, 24]. A maximum likelihood tree with bootstrap support (1,000 bootstraps) was generated in IQTree (WAG+I best-fit model by BIC) [5] based on the TRH2 amino acid sequence alignment and a mid-rooted tree visualized in FigTree v1.4.4 (http://tree.bio.ed.ac.uk/software/figtree/).

Resistome

The assemblies were screened for acquired antimicrobial resistance genes (not point mutations) using default settings in ABRicate (Seemann, T, https://github.com/tseemann/abricate) which uses the NCBI database AMRFinderPlus [25].

Plasmid detection

Assemblies were screened for the presence of plasmids using MOBsuite [26]. A BLAST search was conducted for the RDH3 small plasmid which was not detected by MOBsuite but apparent on an agarose gel and the mapped genome (see 2.6.7).

Genome mapping of genomic islands for RDH3 & M51

Genome mapping was conducted for two genomes (RDH3 and M51) for which Illumina short-read and PacBio long-read data were available. The hybrid assembly pipeline Unicycler (https://github.com/rrwick/Unicycler) (bold bridging mode)[27] and visualization tool Bandage (https://rrwick.github.io/Bandage/)[28] were accessed on the online platform Galaxy Australia (https://usegalaxy.org.au)[29]. A BLAST search was conducted to locate the

virulence genes *vtrB, hlyB* and *trh* on the RDH3 genome (see accession numbers in Virulome section) and to assess the similarity of the plasmid to previously described plasmids. Prokka [30] was used to annotate the genomes which were uploaded to IslandViewer4 (https://www.pathogenomics.sfu.ca/islandviewer/) to map genomic islands (reference genome *Vibrio parahaemolyticus* RIMD 2210633)[31]. Chromosome-2 of RDH3 and M51 were aligned and visualized with AliTV v1.0.6 [32] and default settings using LASTZ as pairwise sequence aligner. Link filtering was set to 500 bp minimum length and 80 to 100% sequence identity. Annotated genes of the pathogenicity island VPaI-β were mapped and visualized using Proksee [33] and mobileOG-db [34] was used to predict mobile genetic elements.

Pangenome analysis

A pangenome of the 77 *V. parahaemolyticus* isolates (29 from this study and 48 global isolates) was constructed in Roary [35] and visualized in Phandango after annotating all assembled genomes with Prokka.

Supplementary Results

Recombination Analysis

See File S1 for details. The homologous recombination analysis showed a mean DNA import length of 170 bp (delta), a mean divergence of the imported DNA of 0.019 (nu) and overall recombination to mutation rate (R/theta) of 0.67 indicating that for every one recombination event, there were 1.5 mutation events. There were several homologous recombination hotspot regions shared between isolates (red bars in Supplementary Figure 1). One major hotspot was on chromosome-1 (reference M51, at 3.01 Mb) containing genes involved in lipopolysaccharide (LPS) biosynthesis (*rfaF, waaA, fdtA-C, rffH*) or biofilm formation (*bcs1*). A second larger hotspot was on chromosome-2 (at 582 kb) with genes involved in metabolic processes such as fatty acid metabolism (*thlA_2, acdA*), the cytochrome bo3 oxidase complex (*cyoB-E*) for aerobic respiration or *sspA*_2 for stress response to starvations.

In contrast to R/theta rates, the ratio r/m relates to the ratio of the actual number of SNPs introduced by recombination over those introduced by mutation. A comparison of r/m across all isolates and nodes showed that for 74% of isolates and 90.0% (125/139) of nodes, the genetic divergence from the reference M51 core genome was mainly driven by vertically inherited mutations with a r/m ratio of 0.04 to 0.42. However, for 13 isolates (and one node), the r/m ratio was large with 4 to 27 suggesting that their genetic divergence from M51 was driven by recombination. These included 7 American isolates, 5 Asian and 1 Australian isolate (M56), all from various sources. They clustered in six distinct clades with 2-3 isolates each separated by only short branch lengths and clade-specific recombination blocks (Figure 2, Supplementary Figure 2). One clade included three isolates of the widespread pandemic strain O3:K6 ST3 which emerged in India in 1996 [36](Figure 2), another clade contained the outbreak strain ST120 (SRR2559309, Peru, oyster) [37] and triple locus variant ST1518 (SRR19513225, Ecuador, shrimps) while a third cluster with a high recombination signal consisted of the above described ST3579 (M56, oyster, Australia) and single-locus variant ST1750 (ERR12422576, fish, China).

Filtering of recombinogenic regions resulted in numerous rearrangements in the broad cluster containing Australian, Asian, and South American isolates reflecting their short internal branch lengths while the topology of the North American cluster remained largely the same pre- and post-filtering (tanglegram in Supplementary Figure 2).

Pangenome

A pangenome analysis of the 77 *V. parahaemolyticus* genomes revealed 23,377 different annotated genes of which only 14.7% (3,443/23,377) were defined as core genome occurring in 99% of genomes with the remaining 85.3% of genes belonging to the accessory genome (Supplementary Figure 3). Close to 80% of genes occurred in less than 15% of genomes. A hierarchical tree based on the presence-absence of all genes showed the

clustering of five of nine genomes which were *trh* and *vtrB* positive and this cluster also contained three of the five genomes with *hlyB* detected. The gastrointestinal strain RDH3, and Australian outbreak related strains SRR17035888 and SRR17673828 [38] were part of this cluster as well as SRR12785886 (oyster, Germany, 2021), SRR19505418 (oyster, Canada, 2016) while ERR12422576 (fish, China, 2020) was on a neighbouring branch (Supplementary Figure 3). These genomes also contained other genes associated with pathogenicity islands including the urease gene cluster and genes encoding the nickel import system as well as T3SS associated genes (*hrcN* and *yscU-2*) (red box in Supplementary Figure 3). The green box contains genes unique to a set of seven isolates which cluster on a long branch. Five of these stem from snails collected at the same time and location in the Darwin region (M24,28-31) and have an identical core and near identical accessory genome. The other two genomes of this cluster belong to unrelated isolates collected from oysters in the remote Daly region, northern Australia (M40) and from Canada (SRR19505386). Accessory genes specific to this cluster encode stress response and potential virulence factors such as the HipA-HipB toxin-antitoxin system, toxin-coregulated pilus (TcpE), disulphide bond formation protein DsbB or DNA repair proteins such as RdgC, RecX. It was of interest that M26 which was collected at the same time and location as M24,28-31 and shared the same core genome, lacked the genes of the green box (Supplementary Figure 3).

Virulome and TRH variants

All isolates from this study were PCR positive for the *V. parahaemolyticus* species marker gene *tlh* and negative for the hemolysin toxin gene *tdh*. The faecal clinical isolate, RDH3, was PCR positive for the thermolabile hemolysin toxin gene *trh*.

In silico screening results are shown in Supplementary Table 3. For the majority of virulence markers, the isolates were either all positive or negative with no variation across isolates. Exceptions were the *trh (trhx)* gene, the alpha hemolysin operon gene *hlyB* and *vtrB* which encodes a ToxR-like transcriptional regulatory protein controlling the expression of pathogenicity island genes [39]. These were detected in the same isolate RDH3 which was also PCR positive for *trh*. The detection of *vtrB* showed 100% coverage (534 bp) and 98.7% identity. In contrast, while *trh* and *hlyB* also had a 100% coverage (570 bp and 2,123 bp length), their percent identity was lower with 86% for *trh* and 82% for *hlyB*. The corresponding database sequences of these genes stemmed from a clinical US *V. parahaemolyticus* strain MAVP-RPI for *vtrB* [40], a clinical *V. parahaemolyticus* strain TH3766 from Japan for *trh* (Genbank AAB29385)[41] while the *hlyB* reference sequence was from *Escherichia coli* CFT073 (Genbank WP_000376545).

The 48 public genomes included in the phylogenetic tree were also screened *in silico* for virulence genes and *tdh* (NP_800824)*, trh, hlyB* and *vtrB* results are shown in Figure 3. The *tdh* gene was detected in five of the 48 public genomes and *trh* in eight public isolates (with 84-85% identity for six and 99-100% for two isolates). *VtrB* was detected in the same eight public isolates while *hlyB* occurred in four of these genomes with 82% identity. The genes *trh* and *vtrB* co-occurred in all instances while if *hlyB* was present, it co-occurred with *vtrB* and *trh*. The presence of *tdh* varied and in only two of five instances, *trh* and *vtrB* were also present. In both of these instances, *trh* showed 99-100% identity i.e. these were SRR17035888 and SRR25745527.

TRH variants and characteristics

Two main *trh* variants have been described in *V. parahaemolyticus* with *trh1* and *trh2* sharing 84% sequence identity [42]. *Trh2* was the variant found in RDH3. The amino acid sequence (189 amino acids – annotated by Prokka) was identical to UniProt TRH2 AOA162S174 from a US clinical strain 4591 [24] with a predicted protein structure based on Alpha-Fold [43]. This was used to predict the protein structure of RDH3 TRH2 which showed 10 beta-strands and one alpha-helix between the seventh and eighth strand (Figure 4). The amino acid sequence was compared to the other eight TRH sequences detected in this study (Figure 4, Supplementary Figure 3A-B). There were two TRH1 sequences of which one was from the Australian clinical isolate SRR17035888 with 100% amino acid sequence identity to the VFDB reference TRHx and the other one was a truncated TRH1 from US SRR25745527 (source unknown) with the last 40 amino acids missing. With the exception of the truncated TRH1, two cysteines were found in all sequences including TRH2 in the C-terminal region (C175, C185) forming a disulphide bond essential for the inter-protein interactions in the tetramer [44, 45]. The remaining seven TRH sequences belonged to the TRH2 group with 89 to 100% amino acid sequence identity amongst them and 84 to 87% identity to TRH1 (SRR17035888) (Supplementary Figure 3A). A phylogenetic tree showed two clusters of TRH2 (Supplementary Figure 3B) with RDH3-TRH2 clustering with SRR12785886 (oysters, Germany) and SRR19513275 (shrimps, Bangladesh) at 95-96% identity. There were four main distinctions in amino acid characteristics between these two TRH2 clusters: at position 38 at the start of the first beta-strand, the RDH3 TRH2 cluster contained a hydrophobic alanine instead of a negatively charged hydrophilic aspartate (A38D); at position 113 between 6th and 7th beta-strands, a negatively charged aspartate instead of a positively charged histidine (D113H); at positions 131-132 at the start of the alpha-helix, hydrophilic tyrosine and serine instead of hydrophobic phenylalanine followed by hydrophilic tyrosine (Y131F, S132Y); and at position 155 between the 8th and 9th beta-strand, a hydrophilic serine instead of non-polar proline with its unusual cyclic structure known to affect protein folding (S155P) [46] (Figure 4). Mutations unique to RDH3-TRH2 (and UniProt TRH2 AOA162S174) included an aromatic tyrosine instead of small serine at position 136 (both hydrophilic)(Y136S); and at the end of the protein, a hydrophobic alanine instead of hydrophilic serine for all other TRH sequences in this study including TRH1 (A187S).

Genomic mapping and islands of clinical RDH3 and environmental M51

Hybrid short and long read assembly of RDH3 resulted in three contigs corresponding to two chromosomes of 3.21 Mb and 1.86 Mb and a small plasmid of 3.8 kb (Supplementary Figure 5). M51 had two contigs of 3.25 Mb and 1.75 Mb with no plasmid. Genomic island predictions revealed a 99-118 kb island on chromosome I in the same region (1.8 - 1.9 Mb) for both isolates (Figure 5A). The island contained predicted transposases, tyrosine recombinase genes (*xerD*) and toxin-antitoxin modules (*yoeB/yafQ/yefM* for RDH3, *relE* in M51). It also encoded several adaptability, stress-response, and metabolic genes, including

for GNAT-family acetyltransferases (*bar*, *yvbK*), efflux transporters (*aaeA/B*), a biofilm-associated pilus gene (*mshD*_1), a redox-related ubiquinone biosynthesis gene (COQ3_1), and O-antigen/LPS sugar biosynthesis enzymes (*wecD*_1/2). The pangenome analysis showed the ubiquitous presence of many of these genes across tested isolates suggesting this to be a common fitness island.

RDH3 had a second 102 kb genomic island on chromosome II which was absent in M51 (Figure 5A-C). This island encoded the three detected virulence genes (*hlyB, trh* and *vtrB*) as well as a urease gene cluster, nickel import system, and some type III secretion system related genes, namely ATP synthase (*hrcN*), effector protein coding *yopJ* gene and *yscU* coding for Yop translocation proteins (Figure 5D). This island is known as pathogenicity island *trh*PAI [47] or VPaI-ß [40]. It also contained a *hns_2* gene with Histone-like Nucleoid Structuring proteins regulating the expression of virulence factors and *pdeG_2*, encoding a putative cyclic di-GMP phosphodiesterase controlling biofilm formation and virulence. There were also several predicted transposases including one flanking the *trh* gene.

Plasmid detection

A plasmid was detected in four of the 29 isolates. Three of these were from clinical isolates (RDH3-5) and one from a diseased fish (M132) (Table 1).

The small plasmid (3.8 kb) in RDH3 was not detected by MOB-suite but was apparent on an agarose gel of the genomic DNA and a hybrid assembly revealed the plasmid (see previous section). A BLAST search showed it had 92% identity to the plasmid pSO5Y of the *Vibrio cholerae* strain SO5Y (CP089144, 3.5 kb) for 55% of its length while for another 48%, it showed 96% identity to the 1.8 kb plasmid pVP-16-VB00198-2 (CP097358.1) of the *V. parahaemolyticus* strain 16-VB00198 (Germany, oyster, 2022). Genome annotation also predicted a cold shock-like protein CspG gene in the latter region.

A contig (length 79.5 kb) of the RDH4 isolate had high similarity (mash distance 0.022, average nucleotide identity ANI ~ 97.5%) to the plasmid p0908 (NC_010113)(81.4 kb) of a *Vibrio* spp. from sediment in the USA (1998)[48]. The plasmid p0908 was also reported to contain enterobacteria phage P1 genes. RDH5 had a contig (170.4 kb) which was typed as the larger plasmid pVCGX2 (CP020079) (204 kb) of the *Vibrio campbellii* strain 20130629003S01 identified in China. Gene annotation of these plasmids revealed mainly genes encoding hypothetical or plasmid maintenance proteins with the exception of *pilT* on the RDH5 plasmid which encodes the ATPase component of the type IV pilus – also called twitching motility protein. This amino acid sequence differed from the core genome encoded PilT-1 and -2 and a BLASTp search showed that it was identical to a PilT encoded by a plasmid in *V. alginolyticus*.

Finally, two contigs (combined length 86.5kb) of M132 showed very high similarity to the plasmid pVPUCMV (CP007006)(BLAST search e-value 0, mash distance 0.009, ANI 99.1%)[49]. The 88.5kb plasmid has been described in a *V. parahaemolyticus* UCM-V493 strain cultured from sediment in Spain in 2002. A BLAST search showed no similarity between the M132 plasmid and the pVA plasmid (NZ_CP043423) which was described in *V.*

parahaemolyticus from shrimp which had the same ST (ST2013) as M132 and suffered from acute hepatopancreatic necrosis disease caused by *Photorhabdus* insect-related (Pir) toxin encoded by *pirA* and *B* genes on the pVA plasmid [50]. Instead, Prokka gene annotation identified a secretion system secretin in the M132 plasmid with a 100% match to a T2SS pathway component GspD/PulD secretin by BLASTp.

Antimicrobial resistance & E-test results

In silico screening revealed the presence of beta-lactamase *blaCARB* gene variants in all isolates used in this study (Supplementary Table 4). Similarly, all genomes had the *tet* (34 and 35) genes which can contribute to resistance to tetracyclines. The isolate M132 from a captive barramundi also had the *qnrS5* gene (NG_050546.1) encoding the quinolone resistance pentapeptide repeat protein associated with reduced susceptibility to quinolones. The gene was not located on the M132 plasmid. The FosG/FosC2 family fosfomycin resistance glutathione transferase gene (NG_050560.1) was found in the clinical isolate RDH1. *In silico* screening of the public genomes included in the phylogenetic tree (Figure 2) revealed one more *V. parahaemolyticus* isolate (SRR19513301 – isolated from shrimp in Thailand) with the fosfomycin resistance gene and one isolate (ERR12422576) with the *qnrS5* gene. Similar to M132, ERR12422576 was also isolated from a fish (in China). E-test results showed that all three tested *Vibrio* isolates (RDH1, RDH3 and M132) were susceptible to the two tested fluoro-quinolones ciprofloxacin and norfloxacin with MIC ranging between 0.125 and 0.19 mg/L for M132, RDH1 and 3 and 0.125 to 2 mg/L for *E. coli* and *P. aeruginosa*. While EUCAST susceptibility breakpoints are not defined for fosfomycin and *Vibrio* spp., we still conducted the testing and confirmed a reduced susceptibility of RDH1 to fosfomycin with a MIC of 24 mg/L which compared to 3 mg/L for M132 and 3 and 0.75 mg/L for the *P. aeruginosa* and *E. coli* control isolates. RDH3 also showed a reduced susceptibility to fosfomycin with a MIC of 32 mg/L.

Supplementary Tables

Supplementary Table 1

Source and date of *V. parahaemolyticus* isolates from this study (all environmental isolates are from the Northern Territory, Northern Australia). Source of infection for the clinical isolates RDH1-5 is unknown.

Name	***Source***	***Location***	***Lats/longs***	***Date Isolated***
M18	Seawater	Rapid Ck	-12.411270°/ 130.840313°	10/12/2020
M19	Seawater	Rapid Ck	-12.411270°/ 130.840313°	10/12/2020
M20	Seawater	Rapid Ck	-12.411270°/ 130.840313°	10/12/2020
M21	Seawater	Rapid Ck	-12.411270°/ 130.840313°	10/12/2020
M24	*Telescopium telescopium*	Rapid Ck	-12.411270°/ 130.840313°	10/12/2020
M25	*T. telescopium*	Rapid Ck	-12.411270°/ 130.840313°	10/12/2020
M26	*T. telescopium*	Rapid Ck	-12.411270°/ 130.840313°	10/12/2020
M27	*T. telescopium*	Rapid Ck	-12.411270°/ 130.840313°	10/12/2020
M28	*T. telescopium*	Rapid Ck	-12.411270°/ 130.840313°	10/12/2020
M29	*T. telescopium*	Rapid Ck	-12.411270°/ 130.840313°	10/12/2020
M30	*T. telescopium*	Rapid Ck	-12.411270°/ 130.840313°	10/12/2020

M31	*T. telescopium*	Rapid Ck	-12.411270°/ 130.840313°	10/12/2020
M35	*Saccostrea mordax*/lineage A	West Daly	-14.19640°/ 129.44356°	8/3/2021
M36	*Saccostrea mordax*/lineage A	West Daly	-14.19640°/ 129.44356°	8/3/2021
M37	*Saccostrea mordax*/lineage A	West Daly	-14.19640°/ 129.44356°	8/3/2021
M38	*Saccostrea mordax*/lineage A	West Daly	-14.19640°/ 129.44356°	8/3/2021
M39	*Saccostrea mordax*/lineage A	West Daly	-14.19640°/ 129.44356°	8/3/2021
M40	*Saccostrea mordax*/lineage A	West Daly	-14.19640°/ 129.44356°	8/3/2021
M51	*Saccostrea spathulata*	Tiwi Is	-11.34097°/ 130.23645°	16/3/2021
M56	*Saccostrea spathulata*	Tiwi Is	-11.34097°/ 130.23645°	16/3/2021
M116	*Saccostrea mordax*/lineage A	Buffalo Creek	-12.338155°/130.908322°	10/5/2021
M117	*Saccostrea mordax*/lineage A	Buffalo Creek	-12.338155°/130.908322°	10/5/2021
M118	*Saccostrea mordax*/lineage A	Buffalo Creek	-12.338155°/130.908322°	10/5/2021
M132	*Lates calcarifer* (Barramundi) (captive)	Darwin region		16/2/2022
Vp RDH1	Clinical wound	Territory Pathology		2022
Vp RDH2	Clinical wound	Territory Pathology		2022
Vp RDH3	Clinical faecal	Territory Pathology		2022

Vp RDH4	Clinical wound	Territory Pathology		2022
Vp RDH5	Clinical wound	Territory Pathology		2022

Supplementary Table 2

List of 48 public *V. parahaemolyticus* genomes added to the phylogenetic tree.

**Accession number**	**Source**	**Country**	**Year**
ERR12422491	Fish	China	2020
ERR12422576	Fish	China	2020
ERR5319192	Clinical	Japan	1980
SRR10738127	Seawater	USA	2007
SRR11823775	Seawater	Vietnam	2008
SRR12395869	Seawater	USA	2020
SRR12785886	Oysters	Germany	2021
SRR13023665	Seawater	China	2018
SRR13023679	Seawater	China	2018
SRR14267330	Oysters	China	2022
SRR14267336	Oysters	China	2022
SRR16348888	Seawater	China	2021
SRR17035888	Clinical	Australia	2021
SRR17673828	Clinical	Australia	2021
SRR19505386	Oysters	Canada	2017
SRR19505405	Clams	Canada	2016
SRR19505418	Oysters	Canada	2016
SRR19505432	Clams	Canada	2014
SRR19505444	Clams	Canada	2015
SRR19513211	Shrimps	Cuba	2016
SRR19513213	Shrimps	Thailand	NA
SRR19513215	Shrimps	Thailand	2016
SRR19513216	Shrimps	Vietnam	2016
SRR19513220	Shrimps	Vietnam	2016
SRR19513225	Shrimps	Ecuador	2009
SRR19513226	Shrimps	India	2015
SRR19513234	Shrimps	Vietnam	NA

SRR19513235	Shrimps	Bangladesh	2019
SRR19513238	Shrimps	Ecuador	2018
SRR19513245	Shrimps	Thailand	2017
SRR19513275	Shrimps	Bangladesh	2012
SRR19513301	Shrimps	Thailand	2009
SRR19544258	Mussels	Canada	NA
SRR19544266	Oysters	Canada	2016
SRR21252248	Clinical	Thailand	2015
SRR21403917	Shrimps	Thailand	NA
SRR23930239	NA	USA	NA
SRR24660296	Food	Spain	2021
SRR25320409	Seawater	USA	2010
SRR2559309	Oysters	Peru	2015
SRR25745527	NA	USA	NA
SRR26183087	Oysters	USA	2023
SRR26919725	Oysters	USA	2023
SRR26919726	Oysters	USA	2023
SRR27606216	Oysters	USA	2022
SRR27606226	Oysters	USA	2022
SRR5071106	Clinical	Bangladesh	1999
SRR5074562	Fish	China	2015

Supplementary Table 3

Virulence genes detected in *V. parahaemolyticus* genomes from this study using PCR, Abricate (VFDB database) and SRST2. Only genes are shown which were detected in at least one genome. Coverage was >99% for all (95% for *vscP* and M21, M40, M56) and identity >95% with the exception of *vcrV* being 93% for M25 and RDH3; and *trh* 86% and *hlyB* 82% for RDH3. Red (PCR) or blue (*in silico* VFDB database) indicates gene presence and no colour is gene not detected. Green means novel MLST ST and yellow an ST which has previously been submitted to PubMLST.

	tlh PCR	*trh* PCR	*tdh* PCR	*exsA*	*exsD*	*hlyB*	*MAM7*	*sycN*	*tlh*	*toxR*	*toxS*	*trhX*	*tyeA*	*vcrD*	*vcrG*	*vcrH*	*vcrR*	*vcrV*	*vecA*	*virG*	*vopB*	*vopD*	*vopN*	*vopQ*	*vopR*	*vopS*	*VP1611*	*VPA0450*	*VpadF*	*vscB*	*vscC*	*vscD*	*vscF*	*vscG*	*vscH*	*vscI*	*vscJ*	*vscK*	*vscL*	*vscN*	*vscO*	*vscP*	*vscQ*	*vscR*	*vscS*	*vscT*	*vscU*	*vscX*	*vscY*	*vtrB*	*vxsC*	ST
M18																																																			3584
M19																																																			3590
M20																																																			3578
M21																																																			3580
M24																																																			3581
M25																																																			2249
M26																																																			3581
M27																																																			3588
M28																																																			3581
M29																																																			3581
M30																																																			3581
M31																																																			3581
M35																																																			3582
M36																																																			3586
M37																																																			3586
M38																																																			3586
M39																																																			2058
M40																																																			3576
M51																																																			3574

M56																																																					3579
M116																																																					3577
M117																																																					3575
M118																																																					3583
M132																																																					2013
RDH1																																																					3589
RDH2																																																					3587
RDH3																																																					2901
RDH4																																																					3585
RDH5																																																					2014

Supplementary Table 4

Antimicrobial resistance genes detected in *V. parahaemolyticus* genomes from this study using Abricate (subset of NCBI AMRFinderPlus database). Only genes are shown which were detected in at least one genome. Coverage was >99% for all and identity >97% with the exception of *tet(34)* with an identity of 83%. ND not detected. Red shaded cells indicate the presence of the gene while blank marks not detected.

	Antimicrobial resistance predicted for														
	fosfomycin	quinolone	beta-lactams											tetracycline	
Isolate	fosA-491618165	qnrS5	blaCARB-18	blaCARB-20	blaCARB-24	blaCARB-26	blaCARB-27	blaCARB-29	blaCARB-31	blaCARB-33	blaCARB-35	blaCARB-36	blaCARB-44	tet(34)	tet(35)
M18										■				■	■
M19					■									■	■
M20						■								■	■
M21											■			■	■
M24					■									■	■
M25									■					■	■
M26					■									■	■
M27						■								■	■
M28					■									■	■
M29					■									■	■
M30					■									■	■
M31					■									■	■
M35													■	■	■
M36						■								■	■
M37						■								■	■
M38						■								■	■
M39			■											■	■
M40									■					■	■
M51										■				■	■
M56								■						■	■
M116								■						■	■
M117			■											■	■

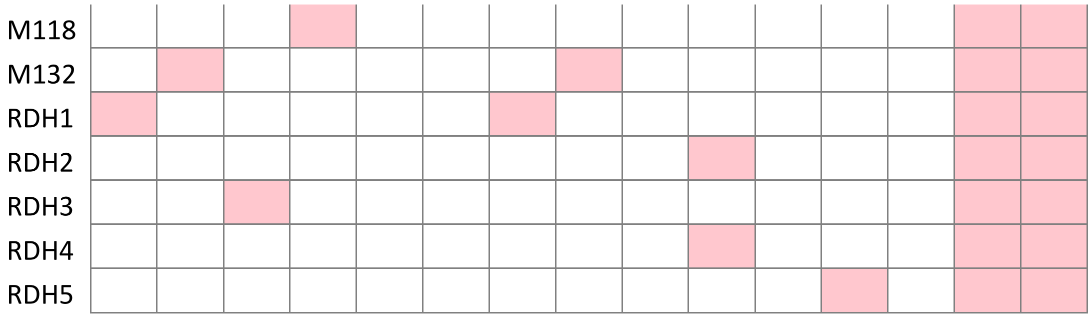
M118
M132
RDH1
RDH2
RDH3
RDH4
RDH5

Supplementary Figures

Supplementary Figure 1

Core genome with recombinations shared by isolates through common ancestry (internal branches) in red. Blue bars mark recombinations unique to individual isolates on terminal branches. Clinical isolates from this study are labelled and the reference M51. Sample source colours: "red" clinical; "dark red" food; "yellow" fish; "green" shellfish; "blue" seawater; "white" unknown; Continent colours: "pink" Americas; "orange" Asia; "blue" Australia; "grey" Europe; The bottom line graph shows the distribution of recombinations across the genome. Recombination predictions and filtered ML tree were generated in Gubbins (and RaxML) and visualized in Phandango.

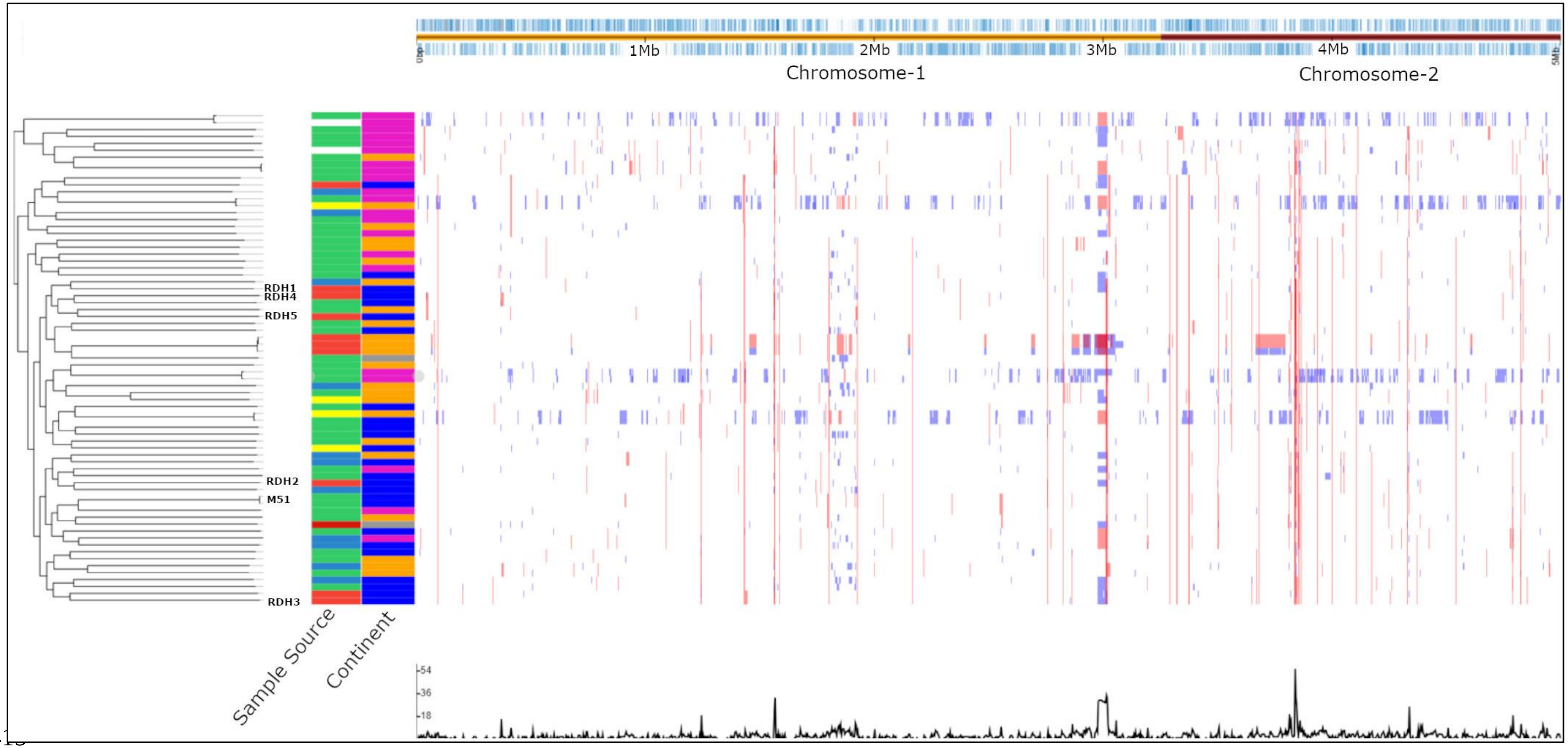

0bp
1Mb
2Mb
3Mb
4Mb
5Mb
Chromosome-1
Chromosome-2
RDH1
RDH4
RDH5
RDH2
M51
RDH3
Sample Source
Continent
54
36
18

Supplementary Figure 2

Comparison of two core genome mid-rooted ML trees with the left the original tree (Supplementary Figure 1) and the right based on recombination filtered sites using ClonalFrameML (Figure 2). Due to the visualization requiring ultrametric dendrograms, the trees were converted to chronograms using a correlated substitution rate model. The label colours indicate the continent of origin with pink Americas, orange Asia, blue Australia and grey Europe. The first letters of the labels mark the source (e.g. Oy oyster, Wat seawater, Clin clinical) while the numbers are the last 4 digits of the genome accession numbers or the ID of the isolates of this study.

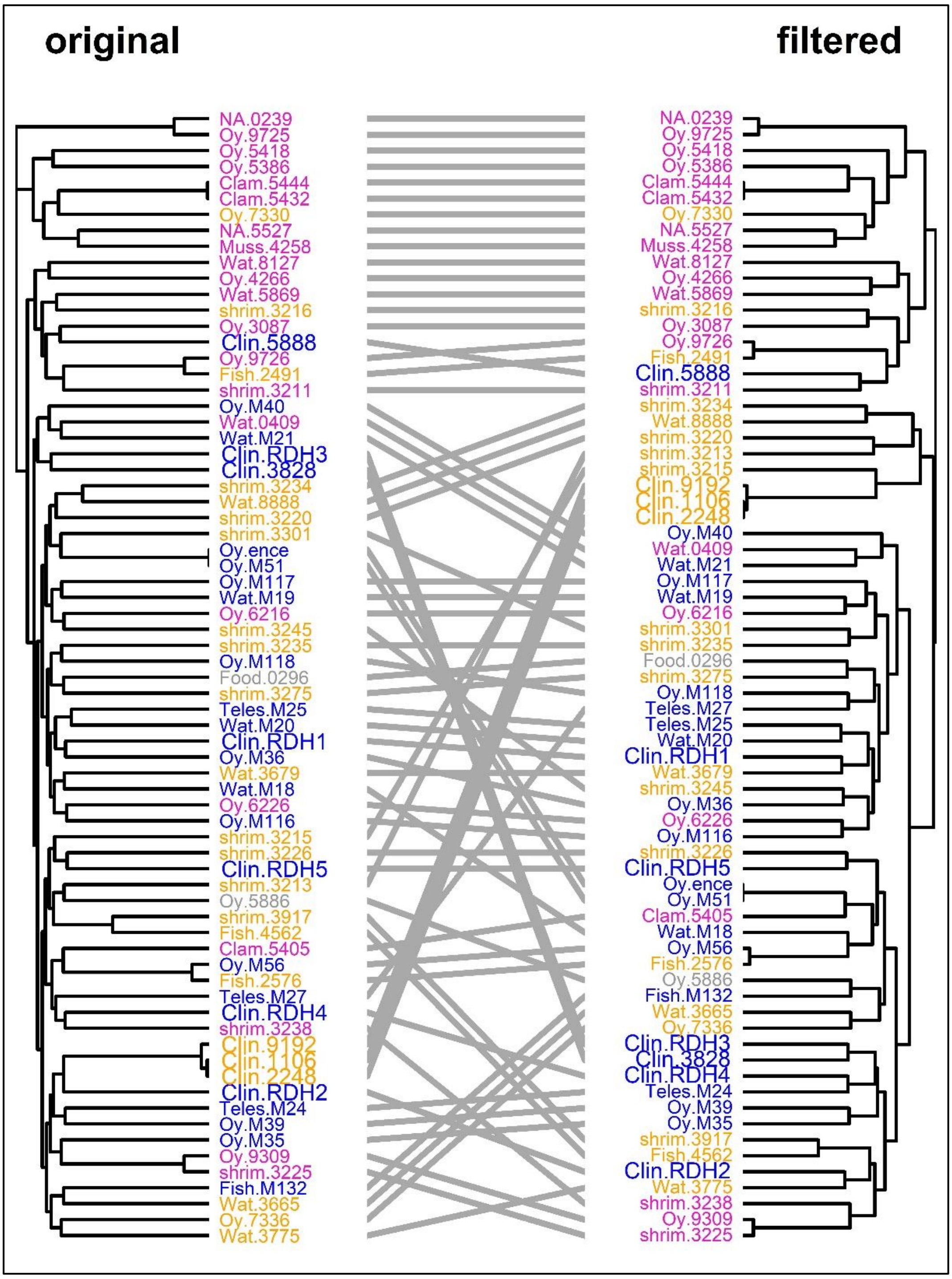

Supplementary Figure 3

Pangenome of 77 V. parahaemolyticus *isolates generated by Roary and visualized in Phandango. The tree is a hierarchical tree based on the presence-absence (P/A) of all genes and given core genes occur in 99% of isolates, clusters of isolates reflect a more similar accessory gene fingerprint. Each column of the blue matrix to the right is a gene which either occurs in an isolate (blue) or is absent (white). The plot at the bottom shows the frequency (%) a gene occurs across the isolates. The red box marks a set of genes which occurred in the* trh/vtrB *positive isolates and included the urease gene cluster, T3SS associated genes, and nickel import system (see also section 3.5). The green box with genes occurring in a set of shellfish isolates included genes encoding proteins for stress response, potential virulence factors or DNA repair. Please see Figure 2 for the colour legend of sample source. Continent blue mark isolates from Australia, pink America, orange Asia, and grey Europe. Isolates of this study are labelled as well as a subset of the public genomes (S and the last four numbers of the SRA accession number – please see Supplement Table 2 for details).*

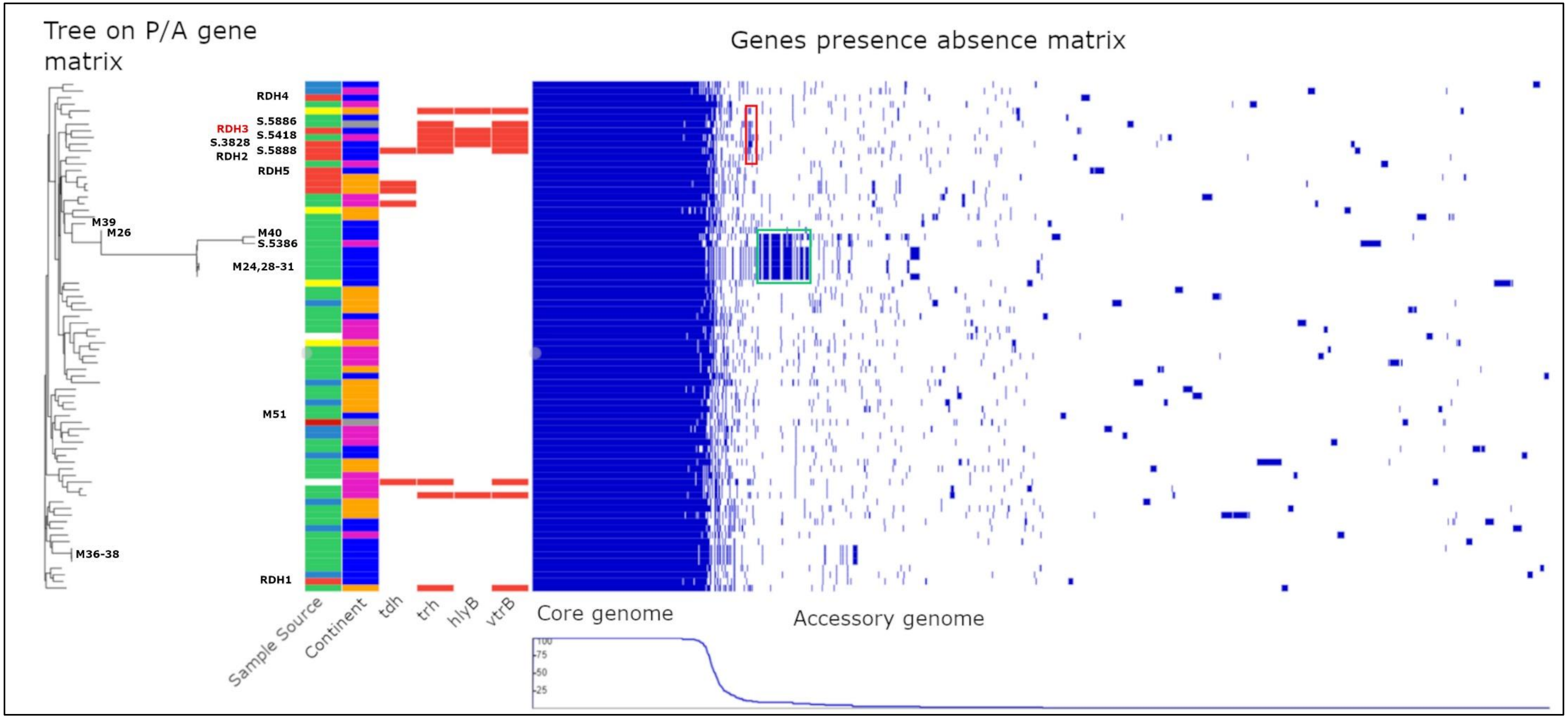
Tree on P/A gene matrix
Genes presence absence matrix
RDH4
RDH3
S.5886
S.3828
S.5418
RDH2
S.5888
RDH5
M39
M26
M40
S.5386
M24,28-31
M51
M36-38
RDH1
Sample Source
Continent
tdh
trh
hlyB
vtrB
Core genome
Accessory genome
100
75
50
25

Supplementary Figure 4

A) *Multiple amino acid sequence alignment of nine TRH variants of this study and three TRH reference sequences (VFDB TRH-X AAB29385; UniProt TRH-1 A0A162SI74, TRH-2 A0A162SI74). The colours of the top alignment show the hydrophobicity of amino acids (most hydrophobic red, most hydrophilic blue) while the bottom (identical) alignment has the amino acids coloured by Zappo (see legend in figure). The AlphaFold predicted protein structure for the RDH3 TRH2 variant is shown bottom left and included 10 beta-strands (green arrows) and one alpha-helix (red) with the first 24 amino acids representing the signal sequence [44]. Figure generated in Jalview 2.11.4.1 [23, 24].* ***B)*** *Matrix showing the number of differing amino acids between 12 TRH variants (total length 189 amino acids) based on a multiple MAFFT amino acid sequence alignment [22] of nine TRH variants of this study and three TRH reference sequences (VFDB database TRH-X sequence AAB29385; UniProt A0A162SI74 TRH-1 variant, and UniProt A0A162SI74 TRH-2 variant)* ***C)*** *Mid-rooted maximum likelihood tree generated with IQTree (WAG+I best-fit model by BIC) [5] based on the MAFFT alignment and visualized in FigTree (http://tree.bio.ed.ac.uk/software/figtree/). Node numbers indicate bootstrap support (1,000 bootstraps).*

A)

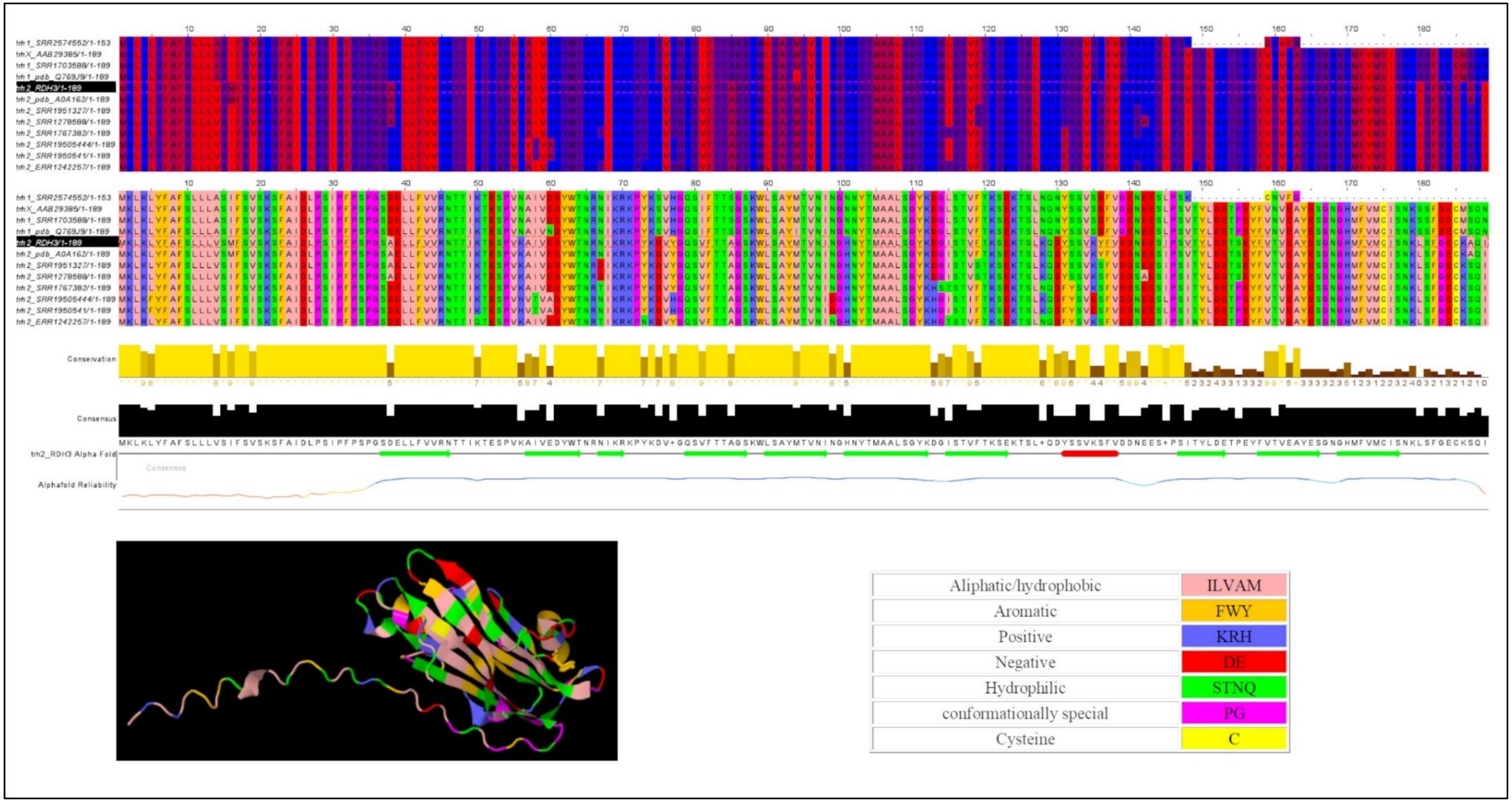
Conservation
Consensus
trh2_RDH3 Alpha Fold
Alphafold Reliability
Aliphatic/hydrophobic
ILVAM
Aromatic
FWY
Positive
KRH
Negative
DE
Hydrophilic
STNQ
conformationally special
PG
Cysteine
C

B)

	trh2_ERR12422576	trh2_SRR17673828	trh2_RDH3	trh2_pdb_A0A162SI74	trh2_SRR12785886	trh2_SRR19513275	trh2_SRR19505418	trh2_SRR19505444	trh1_SRR17035888	trhX_AAB29385	trh1_pdb_Q769J9	trh1_SRR25745527
trh2_ERR12422576	0	6	18	18	16	14	18	18	31	31	32	65
trh2_SRR17673828	6	0	14	14	12	10	16	16	27	27	28	62
trh2_RDH3	18	14	0	0	9	7	20	20	25	25	26	59
trh2_pdb_A0A162SI74	18	14	0	0	9	7	20	20	25	25	26	59
trh2_SRR12785886	16	12	9	9	0	2	21	21	29	29	30	62
trh2_SRR19513275	14	10	7	7	2	0	19	19	27	27	28	61
trh2_SRR19505418	18	16	20	20	21	19	0	0	29	29	30	64
trh2_SRR19505444	18	16	20	20	21	19	0	0	29	29	30	64
trh1_SRR17035888	31	27	25	25	29	27	29	29	0	0	2	40
trhX_AAB29385	31	27	25	25	29	27	29	29	0	0	2	40
trh1_pdb_Q769J9	32	28	26	26	30	28	30	30	2	2	0	42
trh1_SRR25745527	65	62	59	59	62	61	64	64	40	40	42	0

C)

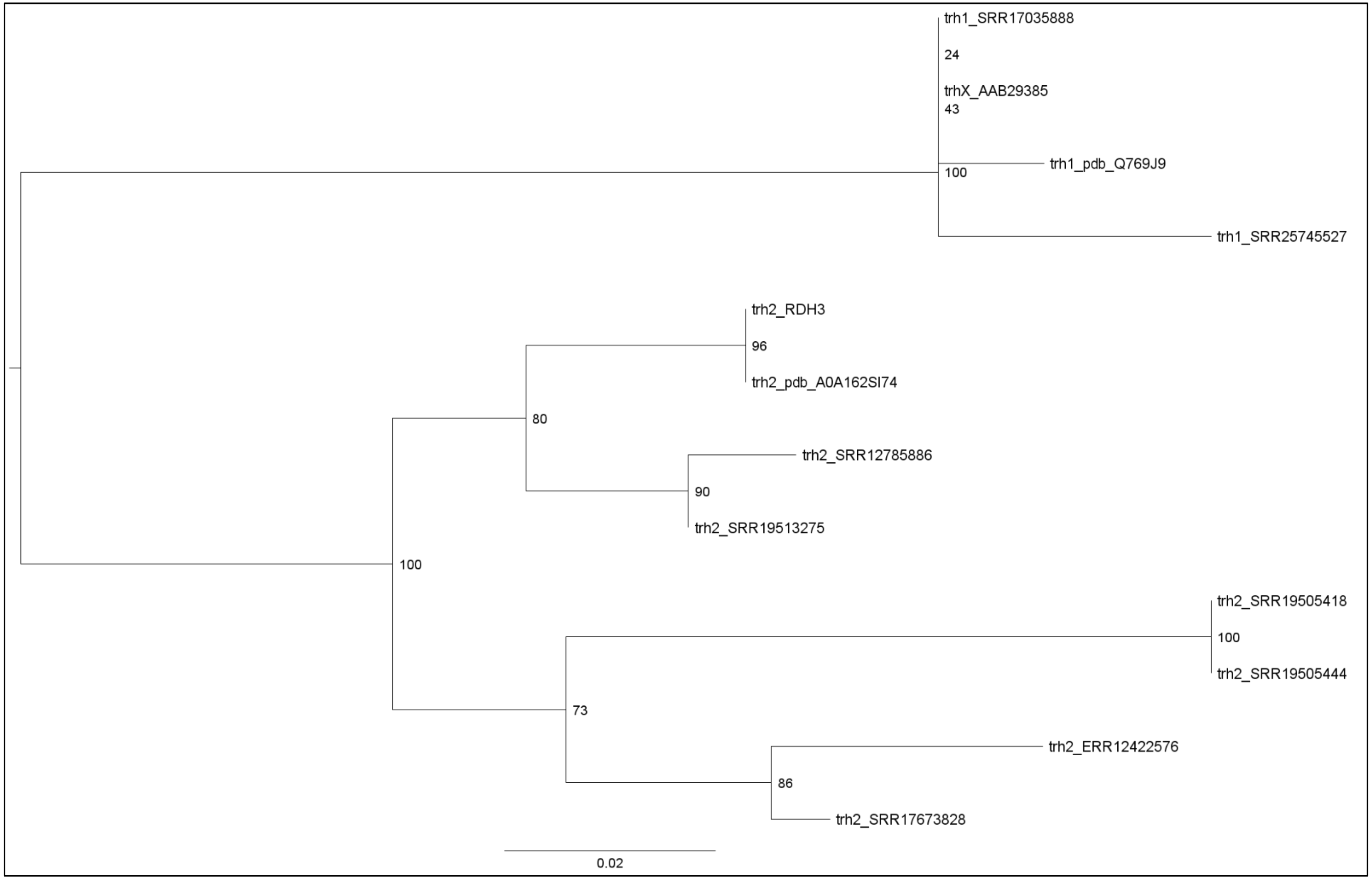
trh1_SRR17035888
24
trhX_AAB29385
43
trh1_pdb_Q769J9
100
trh1_SRR25745527
trh2_RDH3
96
trh2_pdb_A0A162SI74
80
trh2_SRR12785886
90
trh2_SRR19513275
100
trh2_SRR19505418
100
trh2_SRR19505444
73
trh2_ERR12422576
86
trh2_SRR17673828
0.02

Supplementary Figure 5

Using Unicycler to assemble the short and long read data of RDH3 followed by Bandage to visualize the assemblies, the two V. parahaemolyticus chromosomes 1 and 2 (still open due to some unresolved repeated sequences) as well as the small plasmid were revealed. The small plasmid was not detected with just using PacBio long read data.

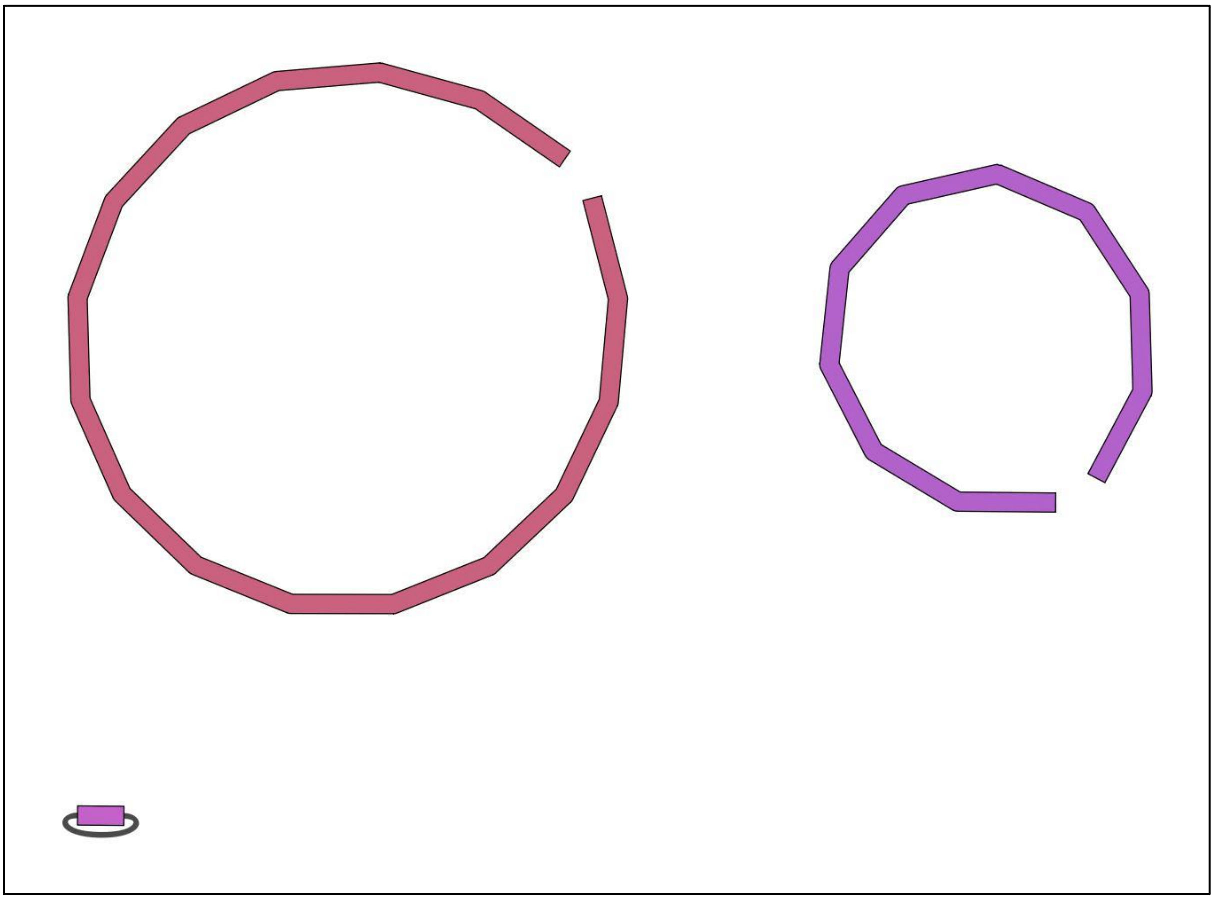

References

1. **Nordstrom JL, Vickery MCL, Blackstone GM, Murray SL, DePaola A**. Development of a Multiplex Real-Time PCR Assay with an Internal Amplification Control for the Detection of Total and Pathogenic <i>Vibrio parahaemolyticus</i> Bacteria in Oysters. *Applied and Environmental Microbiology* 2007;73(18):5840-5847.

2. **Goh SH, Potter S, Wood JO, Hemmingsen SM, Reynolds RP et al.** HSP60 gene sequences as universal targets for microbial species identification: studies with coagulase-negative staphylococci. *Journal of clinical microbiology* 1996;34(4):818-823.

3. **Andrews S**. *FastQC: a quality control tool for high throughput sequence data*. Cambridge, United Kingdom; 2010.

4. **Sarovich DS, Price EP**. SPANDx: a genomics pipeline for comparative analysis of large haploid whole genome re-sequencing datasets. *BMC Research Notes*, journal article 2014;7(1):618.

5. **Nguyen L-T, Schmidt HA, von Haeseler A, Minh BQ**. IQ-TREE: A Fast and Effective Stochastic Algorithm for Estimating Maximum-Likelihood Phylogenies. *Molecular Biology and Evolution* 2014;32(1):268-274.

6. **Kalyaanamoorthy S, Minh BQ, Wong TKF, von Haeseler A, Jermiin LS**. ModelFinder: fast model selection for accurate phylogenetic estimates. *Nature Methods* 2017;14(6):587-589.

7. **Minh BQ, Nguyen MAT, von Haeseler A**. Ultrafast Approximation for Phylogenetic Bootstrap. *Molecular Biology and Evolution* 2013;30(5):1188-1195.

8. **Didelot X, Wilson DJ**. ClonalFrameML: Efficient Inference of Recombination in Whole Bacterial Genomes. *PLOS Computational Biology* 2015;11(2):e1004041.

9. **Xu S, Li L, Luo X, Chen M, Tang W et al.** Ggtree: A serialized data object for visualization of a phylogenetic tree and annotation data. *iMeta* 2022;1(4):e56.

10. **Revell LJ**. phytools 2.0: an updated R ecosystem for phylogenetic comparative methods (and other things). *PeerJ* 2024;12:e16505.

11. **Paradis E, Schliep K**. ape 5.0: an environment for modern phylogenetics and evolutionary analyses in R. *Bioinformatics* 2018;35(3):526-528.

12. **Galili T**. dendextend: an R package for visualizing, adjusting and comparing trees of hierarchical clustering. *Bioinformatics* 2015;31(22):3718-3720.

13. **Croucher NJ, Page AJ, Connor TR, Delaney AJ, Keane JA et al.** Rapid phylogenetic analysis of large samples of recombinant bacterial whole genome sequences using Gubbins. *Nucleic Acids Research* 2014;43(3):e15-e15.

14. **Hadfield J, Croucher NJ, Goater RJ, Abudahab K, Aanensen DM et al.** Phandango: an interactive viewer for bacterial population genomics. *Bioinformatics* 2017;34(2):292-293.

15. **Stamatakis A**. RAxML version 8: a tool for phylogenetic analysis and post-analysis of large phylogenies. *Bioinformatics* 2014;30(9):1312-1313.

16. **Wood DE, Lu J, Langmead B**. Improved metagenomic analysis with Kraken 2. *Genome Biology* 2019;20(1):257.

17. **Bradford LM, Carrillo C, Wong A**. Managing false positives during detection of pathogen sequences in shotgun metagenomics datasets. *BMC Bioinformatics* 2024;25(1):372.

18. **Jolley KA, Maiden MCJ**. BIGSdb: Scalable analysis of bacterial genome variation at the population level. *BMC Bioinformatics* 2010;11(1):595.

19. **González-Escalona N, Martinez-Urtaza J, Romero J, Espejo Romilio T, Jaykus L-A et al.** Determination of Molecular Phylogenetics of Vibrio parahaemolyticus Strains by Multilocus Sequence Typing. *Journal of Bacteriology* 2008;190(8):2831-2840.

20. **Chen L, Zheng D, Liu B, Yang J, Jin Q**. VFDB 2016: hierarchical and refined dataset for big data analysis--10 years on. *Nucleic Acids Res* 2016;44(D1):D694-697.

21. **Inouye M, Dashnow H, Raven L-A, Schultz MB, Pope BJ et al.** SRST2: Rapid genomic surveillance for public health and hospital microbiology labs. *Genome Medicine* 2014;6(11):90.

22. **Katoh K, Standley DM**. MAFFT Multiple Sequence Alignment Software Version 7: Improvements in Performance and Usability. *Molecular Biology and Evolution* 2013;30(4):772-780.

23. **Waterhouse AM, Procter JB, Martin DMA, Clamp M, Barton GJ**. Jalview Version 2—a multiple sequence alignment editor and analysis workbench. *Bioinformatics* 2009;25(9):1189-1191.

24. **Jumper J, Evans R, Pritzel A, Green T, Figurnov M et al.** Highly accurate protein structure prediction with AlphaFold. *Nature* 2021;596(7873):583-589.

25. **Feldgarden M, Brover V, Haft DH, Prasad AB, Slotta DJ et al.** Validating the AMRFinder Tool and Resistance Gene Database by Using Antimicrobial Resistance Genotype-Phenotype Correlations in a Collection of Isolates. *Antimicrob Agents Chemother* 2019;63(11).

26. **Robertson J, Nash JHE**. MOB-suite: software tools for clustering, reconstruction and typing of plasmids from draft assemblies. *Microb Genom* 2018;4(8).

27. **Wick RR, Judd LM, Gorrie CL, Holt KE**. Unicycler: Resolving bacterial genome assemblies from short and long sequencing reads. *PLOS Computational Biology* 2017;13(6):e1005595.

28. **Wick RR, Schultz MB, Zobel J, Holt KE**. Bandage: interactive visualization of de novo genome assemblies. *Bioinformatics* 2015;31(20):3350-3352.

29. **Community TG**. The Galaxy platform for accessible, reproducible, and collaborative data analyses: 2024 update. *Nucleic Acids Research* 2024;52(W1):W83-W94.

30. **Seemann T**. Prokka: rapid prokaryotic genome annotation. *Bioinformatics* 2014;30(14):2068-2069.

31. **Bertelli C, Laird MR, Williams KP, Lau BY, Hoad G et al.** IslandViewer 4: expanded prediction of genomic islands for larger-scale datasets. *Nucleic Acids Res* 2017;45(W1):W30-w35.

32. **Ankenbrand MJ, Hohlfeld S, Hackl T, Förster F**. AliTV—interactive visualization of whole genome comparisons. *PeerJ Computer Science* 2017;3:e116.

33. **Grant JR, Enns E, Marinier E, Mandal A, Herman EK et al.** Proksee: in-depth characterization and visualization of bacterial genomes. *Nucleic Acids Research* 2023;51(W1):W484-W492.

34. **Brown CL, Mullet J, Hindi F, Stoll JE, Gupta S et al.** mobileOG-db: a Manually Curated Database of Protein Families Mediating the Life Cycle of Bacterial Mobile Genetic Elements. *Appl Environ Microbiol* 2022;88(18):e0099122.

35. **Page AJ, Cummins CA, Hunt M, Wong VK, Reuter S et al.** Roary: rapid large-scale prokaryote pan genome analysis. *Bioinformatics* 2015;31(22):3691-3693.

36. **Okuda J, Ishibashi M, Hayakawa E, Nishino T, Takeda Y et al.** Emergence of a unique O3:K6 clone of Vibrio parahaemolyticus in Calcutta, India, and isolation of strains from the same clonal group from Southeast Asian travelers arriving in Japan. *Journal of Clinical Microbiology* 1997;35(12):3150-3155.

37. **Gonzalez-Escalona N, Gavilan RG, Toro M, Zamudio ML, Martinez-Urtaza J**. Outbreak of Vibrio parahaemolyticus sequence type 120, Peru, 2009. *Emerging Infectious Diseases* 2016;22(7):1235.

38. **Fearnley E, Leong LEX, Centofanti A, Dowsett P, Combs BG et al.** Vibrio parahaemolyticus Foodborne Illness Associated with Oysters, Australia, 2021-2022. *Emerg Infect Dis* 2024;30(11):2271-2278.

39. **Kodama T, Gotoh K, Hiyoshi H, Morita M, Izutsu K et al.** Two regulators of Vibrio parahaemolyticus play important roles in enterotoxicity by controlling the expression of genes in the Vp-PAI region. *PLoS One* 2010;5(1):e8678.

40. **Xu F, Gonzalez-Escalona N, Drees KP, Sebra RP, Cooper VS et al.** Parallel Evolution of Two Clades of an Atlantic-Endemic Pathogenic Lineage of Vibrio parahaemolyticus by Independent Acquisition of Related Pathogenicity Islands. *Applied and Environmental Microbiology* 2017;83(18):e01168-01117.

41. **Xu M, Iida T, Yamamoto K, Takarada Y, Miwatani T et al.** Demonstration and characterization of simultaneous production of a thermostable direct hemolysin (TDH/I) and a TDH-related hemolysin (TRHx) by a clinically isolated Vibrio parahaemolyticus strain, TH3766. *Infect Immun* 1994;62(1):166-171.

42. **Kishishita M, Matsuoka N, Kumagai K, Yamasaki S, Takeda Y et al.** Sequence variation in the thermostable direct hemolysin-related hemolysin (trh) gene of Vibrio parahaemolyticus. *Applied and Environmental Microbiology* 1992;58(8):2449-2457.

43. **Nilsson WB, Turner JW**. The thermostable direct hemolysin-related hemolysin (trh) gene of Vibrio parahaemolyticus: Sequence variation and implications for detection and function. *Journal of Microbiological Methods* 2016;126:1-7.

44. **Paria P, Chakraborty HJ, Behera BK, Das Mohapatra PK, Das BK**. Computational characterization and molecular dynamics simulation of the thermostable direct hemolysin-related hemolysin (TRH) amplified from Vibrio parahaemolyticus. *Microbial Pathogenesis* 2019;127:172-182.

45. **Kundu N, Tichkule S, Pandit SB, Chattopadhyay K**. Disulphide bond restrains the C-terminal region of thermostable direct hemolysin during folding to promote oligomerization. *Biochemical Journal* 2017;474(2):317-331.

46. **Levitt M**. Effect of proline residues on protein folding. *Journal of molecular biology* 1981;145(1):251-263.

47. **Chen Y, Stine OC, Badger JH, Gil AI, Nair GB et al.** Comparative genomic analysis of Vibrio parahaemolyticus: serotype conversion and virulence. *BMC genomics* 2011;12(1):294.

48. **Hazen TH, Wu D, Eisen JA, Sobecky PA**. Sequence characterization and comparative analysis of three plasmids isolated from environmental Vibrio spp. *Appl Environ Microbiol* 2007;73(23):7703-7710.

49. **Kalburge SS, Polson SW, Crotty KB, Katz L, Turnsek M et al.** Complete Genome Sequence of Vibrio parahaemolyticus Environmental Strain UCM-V493. *Genome Announcements* 2014;2(2):10.1128/genomea.00159-00114.

50. **Yan CZY, Austin CM, Ayub Q, Rahman S, Gan HM**. Genomic characterization of Vibrio parahaemolyticus from Pacific white shrimp and rearing water in Malaysia reveals novel sequence types and structural variation in genomic regions containing the Photorhabdus insect-related (Pir) toxin-like genes. *FEMS Microbiology Letters* 2019;366(17).
